# Harm reduction in Europe: a framework for civil society-led monitoring

**DOI:** 10.1186/s12954-020-00451-7

**Published:** 2021-01-06

**Authors:** Rafaela Rigoni, Tuukka Tammi, Daan van der Gouwe, Eberhard Schatz

**Affiliations:** 1 Correlation – European Harm Reduction Network, Amsterdam, the Netherlands; 2 Finnish Institute for Health and Welfare, Helsinki, Finland; 3 Harm Reduction Network, Trimbos Institute, Utrecht, the Netherlands

**Keywords:** Monitoring and evaluation, Civil society organisations, Harm reduction, Overdose prevention, Europe

## Abstract

**Background:**

Civil society organisations (CSOs) play a vital role in developing and implementing effective measures to reduce the harms of drug use. They are also fundamental actors to monitor and evaluate programmes and policies for improvement. While harm reduction services are subject to monitoring, and international and European indicators exist, a framework for civil society-led monitoring does not exist. This paper analyses the challenges and added values of developing such a framework for the European region.

**Methods:**

Since 2018, a technical working group within Correlation-European Harm Reduction Network (C-EHRN) is developing and revising a monitoring framework, collecting—through National Focal Points—the experience of harm reduction service providers and service users in 34 European countries. The first round of data collection, in 2019, focused on hepatitis C, overdose prevention, new drug trends and civil society involvement in drug policies.

**Results:**

Developing CSO-based harm reduction monitoring is a learning by doing process. Assuring reliability and national representativeness of the data was a central challenge. As most CSOs have little or no experience with monitoring and research and work in a local-based context, the monitoring approach and its indicators were adjusted to the local context in the second round, bringing more in-depth information and helping to improve results’ reliability. While this implied shifting from the initial focus on comparing responses at a national level, the change to collecting qualitative data reflecting local realities of service policies and delivery provides the foundations for a critical appraisal of these realities against European policy goals. This allowed to map discrepancies between official policies and their implementation, as well as identify gaps in and complement data collection from national-level agencies.

**Conclusions:**

By focusing on local experiences regarding the delivery of global and European policy targets, C-EHRN monitoring uses the unique strengths of its CSOs network and generates information that complements the reporting by other monitoring agencies. Data reflecting the CSOs perspective is essential for optimising local planning of service provision and development of effective and respectful drug policies at national and European level. If data quality issues, as well as the sustainability of reporting, are adequately addressed, civil society monitoring can provide excellent added value for the monitoring of harm reduction in Europe.

## Background: the role of civil society in the monitoring of harm reduction

Civil society organisations (CSOs)^1^ is assumed to function as intermediaries between citizens and policymakers. CSOs can act as transmission belts that filter societal preferences and channel them to policymakers. In practice, however, their capacity to effectively interact with policymakers varies considerably [1]. In the field of harm reduction, CSOs play an essential role in developing and implementing effective measures to address the negative consequences of drug use. They work directly for, and with, people who use drugs (PWUD) and have a good understanding of their daily needs.

Due to the provision of low-threshold services, civil society-based harm reduction agencies are usually the first contact point for PWUD. For this reason, a functioning relationship between CSOs and decision-makers is crucial in ensuring that the public policy responds to the actual needs of PWUD. The inside knowledge and information of communities and grass-root organisations are critical in developing adequate drug policies and practice. Currently, however, a constructive and respectful relationship between policymakers and CSOs is missing in several European countries. In these countries, decision-makers may have minimal knowledge about what PWUD need, resulting in a lack of adequate, inclusive policies, based on mutual understanding and real needs.

Civil society is increasingly assuming the role of holding governments and donors, among others, accountable, by engaging in independent monitoring and evaluation of services and programmes [2]. It has long been shown that community monitoring can play an essential role in improving service delivery [3]. Moreover, in combination with advocacy, monitoring tools are crucial strategies to hold governments accountable and to improve the implementation of policies and programmes in line with the needs of PWUD and their environments [4].

## Existing monitoring activities

There are already well-established monitoring activities in the field of drug use and harm reduction, both globally, in Europe and sometimes also at the national level.

In Europe, systematic harm reduction monitoring began in 2006 with the introduction of data reporting forms on health and social responses to drug use by the European Monitoring Centre for Drugs and Drug Addiction (EMCDDA). Since 2007, the agency publishes harm reduction data collected by its 30 National Focal Points (Reitox NFP Network), which include all European Union (EU) countries plus Norway and Turkey. Other data collected by the agency include demand for drug treatment; prevalence and patterns of drug use; health consequences of drug use such as infectious diseases and drug-related deaths. The core publications of the agency are the European Drug Report [5] and country profiles. The EMCDDA furthermore conducts separate ad hoc studies and presents European overviews on diverse harm reduction themes as well as European public health guidance relevant to harm reduction in the community and prison. Together with its Focal Points, the agency runs an early warning system to identify newly emerging substances that cause harm and new drug trends and disseminates information regarding the effectiveness of harm reduction interventions in its Best Practice Portal [6].

The current harm reduction monitoring tools of the EMCDDA are well developed and bring much valuable information to policymaking and practice. They have been used to inform EU drug policies, including by providing essential background data to discussions in the EU Horizontal Drugs Group and the evaluation of EU Council Recommendations, Drug Action Plans and Strategies since more than a decade. Nevertheless, EMCDDA datasets do not systematically reflect the perspective of harm reduction CSOs and their service users on availability, accessibility and quality of harm reduction interventions. Therefore, data might not reflect the ‘life experiences’ of people who use drugs and the real consequences of specific drug policies.

At a global level, Harm Reduction International (HRI) has conducted a biannual survey since 2008, publishing its data in the report The Global State of Harm Reduction [7]. Data collection involves a coordinated effort across practitioners, academics, advocates and activists, and provides an independent analysis of the state of harm reduction in the world. Concerning Europe, HRI report brings valuable data on the availability of essential harm reduction services in the region, such as needle syringe exchange (NSP), opiate substitution therapy (OST), drug consumption rooms (DCRs) and harm reduction in prisons. It also includes interventions directed to new psychoactive stimulants (NPS) and responses to overdose, HIV, hepatitis C (HCV) and tuberculosis (TB). Finally, it also reflects on policy developments of harm reduction, as well as advocacy and funding. When it comes to the European Union, the report relies mainly on the data compiled and processed by the EMCDDA, as well as direct information from stakeholders in the countries.

The HRI report offers a great comparative view on harm reduction development in different world regions. It includes epidemiological data from official sources, and it reflects civil society perspectives on harm reduction in a systematic way. Nevertheless, as it is dedicated to the global level, its data can only offer a generic and crude overview per region, without much details on policy implementation and experiences at the service delivery level in each country.

## A new attempt and (new) questions

To fill in the gaps left by current monitoring, and aiming at playing a complementary role, Correlation-European Harm Reduction Network (C-EHRN) started to develop a framework for European civil society-based monitoring in 2018. The network uses an online survey as a monitoring tool to collect the experiences of harm reduction service providers and service users at the ground level. It aims, in the long-term, at improving harm reduction responses and policies in Europe. The first C-EHRN annual report was published in 2019 [8]. This is a separate report, intended to serve as a complementary source of data both for EMCDDA and HRI, as well as to the network members.

The current monitoring tool specifically targets developments in the areas of Hepatitis C (HCV), new drug trends, overdose prevention and civil society involvement in drug policies, themes chosen by the members of the network due to their crucial importance for harm reduction. In terms of HCV, the recent introduction of highly effective interferon-free direct-acting antiviral (DAA) regimens has raised the prospect of dramatically increasing treatment uptake and success rates. It is paramount to know to what extent PWUD are accessing DAA treatment and how harm reduction programmes are approaching new cases of HCV infection [9]. Regarding overdose prevention, people with problem drug use patterns show an overall mortality rate of 1–2% per year and people who use opioids are 5–10 times more likely to die than their peers of the same age and gender in Europe [10]. Effective harm reduction responses to reduce the number of overdoses and deaths have been identified by the EMCDDA [5, 11, 12], and C-EHRN monitoring aims at mapping how far these are being applied in practice. Finally, regarding new drug trends, the continued emergence of new substances and changing patterns of drug use in Europe [5] requires better risk information and higher levels of consumer protection, including comprehensive health responses and the constant adaptation of harm reduction interventions. New approaches to update existing data on new drug trends regularly and frequently have been called for [13], and C-EHRN monitoring aims at contributing with timely data to help improve current efforts.

Developing a framework for civil society-led monitoring of harm reduction can be a challenging process. Despite its importance, very little guidance or analytical reflections can be found in the literature. Most of the available academic research on the monitoring of harm reduction is dedicated to the evaluation of harm reduction services in different countries and settings [14]. Various articles and grey literature are devoted to discussing the best indicators to monitor and evaluate harm reduction services [15, 16]. To our knowledge, however, no article discusses the process of developing a framework for civil society-led monitoring of harm reduction. This paper presents an analysis of the challenges encountered and good practices developed while developing the C-EHRN monitoring approach to benefit other civil society actors engaging in this task. With this paper, thus, we aim to describe and discuss the learning process of developing a civil society-led framework to reflect a civil society perspective on harm reduction in Europe at the service delivery and policy implementation levels. We focus on the added value and the challenges of civil society-led monitoring, emphasising its methodological implications. Our reflections are centred around getting relevant and reliable data from civil society actors and making it useful for policymaking and advocacy.

## Methods: the C-EHRN monitoring framework

The European Harm Reduction Network (C-EHRN) is a European civil society network of organisations and individuals with grassroots expertise in the field of drug use, harm reduction and social inclusion. The network was established in 2004 and is hosted by the Regenboog Groep in Amsterdam, the Netherlands. The European Union provided funding for numerous previous and current projects of the network and has awarded a multi-annual grant for the maintenance of the network for the years 2018–2021 This grant made it possible to strengthen the network and to develop and implement meaningful work, including the collection of information and data and the development of the monitoring tool, besides the organisation of events for capacity building and knowledge exchange and the development of relevant advocacy actions to improve the situation of PWUD. C-EHRN currently counts with over 220 members in virtually all EU Member States and surrounding countries. Most members are organisations providing harm reduction services, while a few are individual members (experts, and other members of the community of people who use drugs). C-EHRN advocates for evidence- and rights-based harm reduction, and for civil society to play a vital role in the development and the implementation of harm reduction interventions and policies.

To complement existing European harm reduction monitoring systems with grass-root level data, C-EHRN began to develop a framework for civil society-led monitoring in 2018. To gather data on the experiences of harm reduction service providers and service users at the ground level, C-EHRN counts with a network of national Focal Points (FPs). The Focal Points are C-EHRN organisational members selected by:

- Their willingness to commit to the network’s principles, mission and vision on national and European level;
- Proven thematic expertise in the field of drug use and harm reduction;
- Connectedness on national and European level; and
- Ability to fulfil the role of an intermediary on a national level.

FPs tasks include being consulted for specific thematic or regional expertise; providing input and information, particularly for the monitoring tool activities, including answering the monitoring questionnaire annually. FPs do not receive financial support to perform their functions. Nevertheless, they count with a few benefits, such as being invited to the annual C-EHRN conference (one scholarship available per country); not paying fees for C-EHRN seminars or training; being able to promote their activities on the network’s website and network’s communication channels, and speaking on behalf of the network on the national level. All FPs sign an agreement with C-EHRN, which can be terminated by both parties at any point. C-EHRN strives to select at least one FP per country, but some countries can have more than one representative if additional thematic expertise is needed. C-EHRN currently counts with 37 FPs for 36 countries (a list of FPs is available in the Additional file 1).

C-EHRN established four expert groups to support the development of the monitoring framework, draft the questionnaires, assess the data and review the final report. These are a scientific expert group (SEG) and three thematic expert groups for HCV, overdose prevention and new drug trends. Members of the SEG were selected among the network membership base. They have a proven track record of expertise in the given field, including in monitoring, and their competencies cover different areas—both civil society and academia. These groups, together with C-EHRN staff, contributed to the development of the framework and the implementation of the first rounds of C-EHRN monitoring by providing input and advice. They also directly added to the formulation of the monitoring questionnaires.

**Fig. 1:**
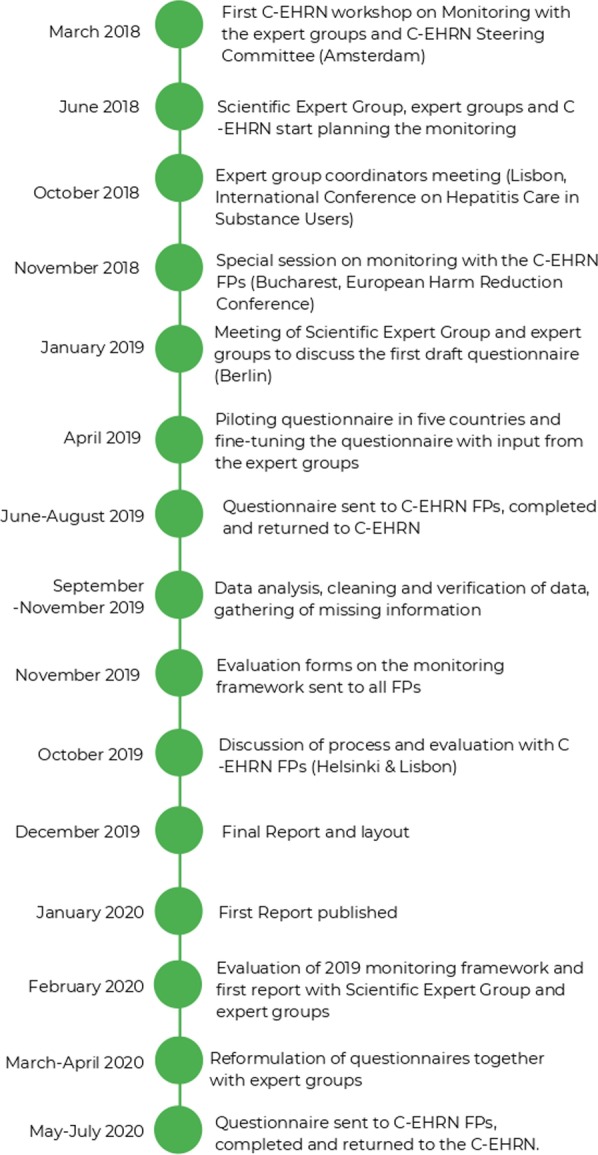
Monitoring framework development timeline

Figure 1 presents a timeline of the development of the C-EHRN monitoring framework.

After finalising the first draft of the monitoring questionnaire in 2018, the tool was piloted in five countries (Finland, Germany, Italy, Poland and Romania) at the beginning of 2019. Based on feedback during the pilot phase, the questionnaire was adapted. The 2019 questionnaire (available as Additional file 2) was composed of 112 questions, divided between the themes of OD prevention, HCV, civil society involvement (CSI) and new drug trends (NDT). All sections contained both single, multiple-choice and open-ended questions. The latter asked for further clarification on closed questions’ choices or for examples and description of experiences. Table 1 shows the main themes investigated and the types of questions used in each section in the 2019 version.

**Table 1:** Main themes investigated in 2019

Domain	Topic	Questions type
*OD prevention*		
Policy level	Existence of OD prevention guidelines or policies (national and local)	Multiple choice (MC)
Naloxone	Availability of naloxone (also in prison and to different groups of PWUD) and access barriers	MC + open ended (OE)
	Availability of take-home naloxone, access barriers, and divergences between official guidelines and practice	MC + OE
	Cost and types of naloxone available	OE
	Availability of naloxone training	MC + OE
	Future plans to increase naloxone	OE
Drug consumption rooms	New initiatives and legal framework to allow for DCRs	MC + OE
Prison settings	Availability of naloxone and pre-release naloxone in prison	MC + OE
	OD prevention upon release	MC
Other measures	Evaluation of first responders	OE
	Groups receiving OD prevention and education	MC + OE
	OF and fentanyl	MC + OE
	OD prevention for non-opioids	MC + OE
*CSI*		
Cooperation with policymakers	Level of cooperation between CSOs and policymakers (country level)	MC + OE
	Level of CSOs satisfaction with the cooperation	OE
Involvement in data collection	FPs contribution to data collection	MC + OE
*HCV*		
National legislation	National legislation and guidelines for HCV treatment	MC
	Perceived impact of the guidelines on service accessibility for PWID	MC
	Availability and restrictions of DAA’s for PWID	MC
	Divergences between official guidelines on DAAs and practice	MC + OE
Continuum of care	Types of services providing testing and treatment for PWID	MC
	Service provider’s investment in prevention, testing or treatment	MC
	PWUD networks participation on HCV advocacy	MC + OE
	Main limitations to address HCV	OE
*NDT*		
	Predominant ‘traditional’ drugs of use (country/region/city)	OE
	Predominant new substances of use (country/region/city)	OE
	Developments in the past 6 months (new substances or new groups of PWUD)	MC + OE
	Description of new substances (name, period, appearance, price, (un) desired effects, etc.	MC + OE
	Description of new groups of PWUD (substances used, forms of use, motives to use)	OE

The questionnaire was distributed among all FPs both as an online survey and as PDF attached to the mail. The PDF was intended as a working document to be shared with contributors to the data gathering. FPs sent the compiled data to C-EHRN through the online survey link. Closed questions were analysed for general percentages or represented in tables with descriptions of features per country. Open-ended responses were analysed with thematic analysis [17] and key findings illustrated with quotes. Data were verified and analysed by C-EHRN staff and an external consultant. The first report [8] was revised by the Scientific Expert Group and the four thematic expert groups.

## Results: Added value and challenges of civil society-led monitoring

Below we describe both the achievements and the challenges of the C-EHRN monitoring tool so far, as they shed light onto the role of civil society in contributing to the production of evidence. As challenges, we call attention to assuring data quality, consistency and reliability, adjusting the monitoring focus to fit CSOs perspectives and competencies, and fine-tuning indicators and methods for data gathering. As achievements, we highlight the added value of CSOs in mapping discrepancies between official policies and policy in practice, identifying gaps in current data collection, and complementing data from official agencies. Finally, we discuss the need to assure that the monitoring results are also directly relevant to the work of CSOs and FPs contributing to data collection.

## Assuring data quality and consistency

One of the pillars of civil society-led monitoring is the reliance on the capability and capacity of CSOs to collect relevant, reliable and timely data. It is fundamental when choosing a Focal Point for implementing monitoring tasks to take into account the experience this CSO might have in data collection and the size and quality of national networks they can draw upon. Most C-EHRN FPs had some previous experience with data collection before partaking in the C-EHRN monitoring tool. Nevertheless, a number of FPs were not directly involved in data collection for monitoring.

Along the first year of development of the C-EHRN monitoring framework, it became clear that FPs differed largely regarding the quality and reliability of data gathered. To level CSOs previous experience, C-EHRN improved and in fact deepened the guidelines and instructions on how to collect data. Specific sessions for FPs were organised during the International Harm Reduction Conference in Porto, in April 2019, the Lisbon Addictions Conference in October 2019 and a follow-up monitoring meeting in Helsinki, December 2019, to discuss and improve the monitoring questionnaire and discuss data gathering methods. In 2020, group and individual online Q&A sessions were offered to FPs. These preparations were perceived as helpful by FPs.

In some cases, data reflected the observations of just one person. In contrast, in other cases the answers were the result of a consultation of various local, regional or national experts. Most FPs invited external experts with specific knowledge on HCV or overdose (OD), for instance, to provide the information they did not know. On average, FPs consulted 5 experts external to their organisation (varying between 1 and 13). Gathering data from these external experts on time and transferring them into a sound answer was regarded as difficult by 67% of the FPs. At times, the experts questioned the validity and purpose of the requests, leaving FP staff to feel as though the endeavour was not legitimate. Based on that, the Monitoring Survey 2020 provided FPs with a signed introduction letter to help with legitimacy when requesting external advice. The letter explains the importance of the monitoring tool and the role of FPs in gathering data.

Given the limitations in terms of numbers of FPs that are able to represent a whole nation, it was emphasised that the primary purpose was not to prepare a representative data collection. Instead, it was to provide a well-grounded critical assessment of the current local situation and recent developments in their harm reduction environments, respecting the level FPs work in.

## Adjusting monitoring to focus on Civil Society’s expertise

C-EHRN’s first framework of the monitoring tool assumed that its National Focal Points would be able to reflect and provide accurate and reliable data on the harm reduction situation in their countries at national level. Nevertheless, after the first round, it became clear that this assumption was incorrect. Many FPs found it difficult to give reliable answers to a number of questions referring to the national level, as their activities take place at city level, and local settings often do not comply with the situation at national level. That resulted in less reliable national data, and in some cases, even after checking and validation by acknowledged experts, data quality was regarded as not suitable for publication. When faced with questions related to the national level for which they did not have enough knowledge, a few FPs turned to official sources to gather the requested data. That not only led to extra work for them but was also counterproductive to the main objective of the C-EHRN monitoring, namely to reflect a civil society perspective and ‘couleur locale’.

The first round of the monitoring also revealed that the national-focused framework was hindering another main objective of the tool, which is reflecting fundamental qualitative data at service delivery level. If C-EHRN FPs are not capable of providing programme level quantitative data and aggregation at the national level, they are most valuable in providing qualitative data on policy implementation at local level. For these reasons, we have decided to shift the focus in the second year (2020) to the local level of the city where the FP is based and to qualitative data. We learned that in order to profit from CSOs expertise, civil society-led monitoring needs to focus as much as possible on the experiences at the local level (of which the CSOs have detailed and reliable information). CSOs are often the first contact point for PWUD for help, care or support and have a good understanding of their daily problems and needs. They have inside knowledge about to what extent official policies get implemented on the ground, and what the bottlenecks are when they do not. The civil society view complements or is sometimes opposed to the perspectives of official governmental agencies. Such knowledge is crucial to feed drug policies and practice, and to serve the needs of PWUD.

The 2020 questionnaire (available as Additional file 3) was formulated to reflect these changes. All sections were fine-tuned to balance national and local level information as well as qualitative and quantitative data. More questions now focus on the implementation (local) level, and the experiences of FPs and their clients. If, on the one hand, the monitoring loses in its ability to reflect a broader European situation focusing on developments at the national level, it gains in reflecting fundamental qualitative data on service delivery level that can only be collected by CSOs, and which is lacking in most national reports.

## Fine-tuning indicators and methods for data gathering

Defining the best workable indicators and methods to gather data was (and continues to be) a challenge in the C-EHRN civil society-led monitoring development. It is out of scope of this paper to present and discuss all indicators assessed in the tool in comparison with the indicators used by other agencies, but it is possible to point at a few directions. Priority indicators to monitor the coverage and quality of harm reduction services have been previously developed, in collaboration with several experts and CSOs [16]. Such indicators focus on OST (including ‘coverage’, ‘waiting list time’, ‘dosage’ and ‘availability in prisons’) and NSP programmes (‘coverage’, ‘number of needles/syringes distributed/collected’, ‘provision of other drug use paraphernalia’ and ‘availability in prisons’). They are monitored based on yes/no responses and quantifications. These indicators are crucial in providing programme level quantitative data and aggregation at the national level, allowing for systematic comparable overview across countries. Nonetheless, they may miss other crucial information such as: (1) specific needs and challenges faced by service providers to implement official policies on the ground (e.g. how to increase/assure OST and NSP equality of access when such programmes are available), (2) the emerging needs of new groups of people who use drugs (as non-opioids and non-injecting drug users), (3) the involvement of CSOs in the development and evaluation of policies and services, or (4) the continuum of care and the integration of services assisting PWUD, and (5) developments in other crucial harm reduction interventions such as DCRs, drug checking, OD prevention training and campaigns, among others. This is where C-EHRN monitoring is already bringing fundamental added value. Developing the most suitable indicators and methods to gather this local level and quality-reach data, however, requires time [16].

Perhaps, a better method to gather more in-depth and nuanced qualitative data would be the use of Focus Group Discussions (FGD) and in-depth interviews with representatives from user communities and with acknowledged experts. That was, as a tryout for some domains, already agreed upon, and many FPs had started preparing this for piloting on the second round of the monitoring (2020). Unfortunately, both the COVID-19 pandemic and limited staff capacity represented obstacles for this switch. Besides, it would be virtually impossible to cover all topics currently addressed by the tool solely through interviews and FGDs. As we currently evaluate, a hybrid model might be a good compromise. Mapping discrepancies between official policies and real-life practice.

## Mapping discrepancies between official policies and policies in practice

Despite the challenges, the C-EHRN monitoring has provided already some valuable contributions; however, it is out of scope of this paper to address contributions in all the domains currently covered by the tool. Therefore, as an illustration, we present some examples of contributions brought by data gathering on OD prevention. The non-exhaustive examples were chosen as illustrations of where a civil society-led monitoring tool can add value.

In the C-EHRN monitoring tool, an essential contribution from CSOs was mapping discrepancies between official policies and policy in practice. This can be illustrated by data gathered on the OD-related context and interventions at a local level. Due to FPs close connection to the field, the data gathered were able to map the mismatch between European policy targets, international guidelines/strategies on OD prevention and the real-life situation experienced at local, regional and national levels. One example concerns the presence of take-home naloxone (THN) programmes in European countries.

Drug overdose (OD) deaths have risen consecutively in Europe for the past 5 years. Approximately 80–90% of the overdoses occurring in Europe are linked to the use of opioids [5]. Naloxone is a lifesaving medication for opioid OD reversal which has been recommended by the World Health Organization be available to all people likely to witness an OD: medical emergency staff, staff from harm reduction programmes, and PWUD and their friends/family members [18]. Such wide availability of naloxone can only be reached via community-based take-home naloxone (THN) programmes [19, 20]. Take-home naloxone programmes are increasingly implemented in Europe [17], but not much is known about the actual availability of naloxone ‘in the streets’, i.e. close to those who may need it.

**Fig. 2:**
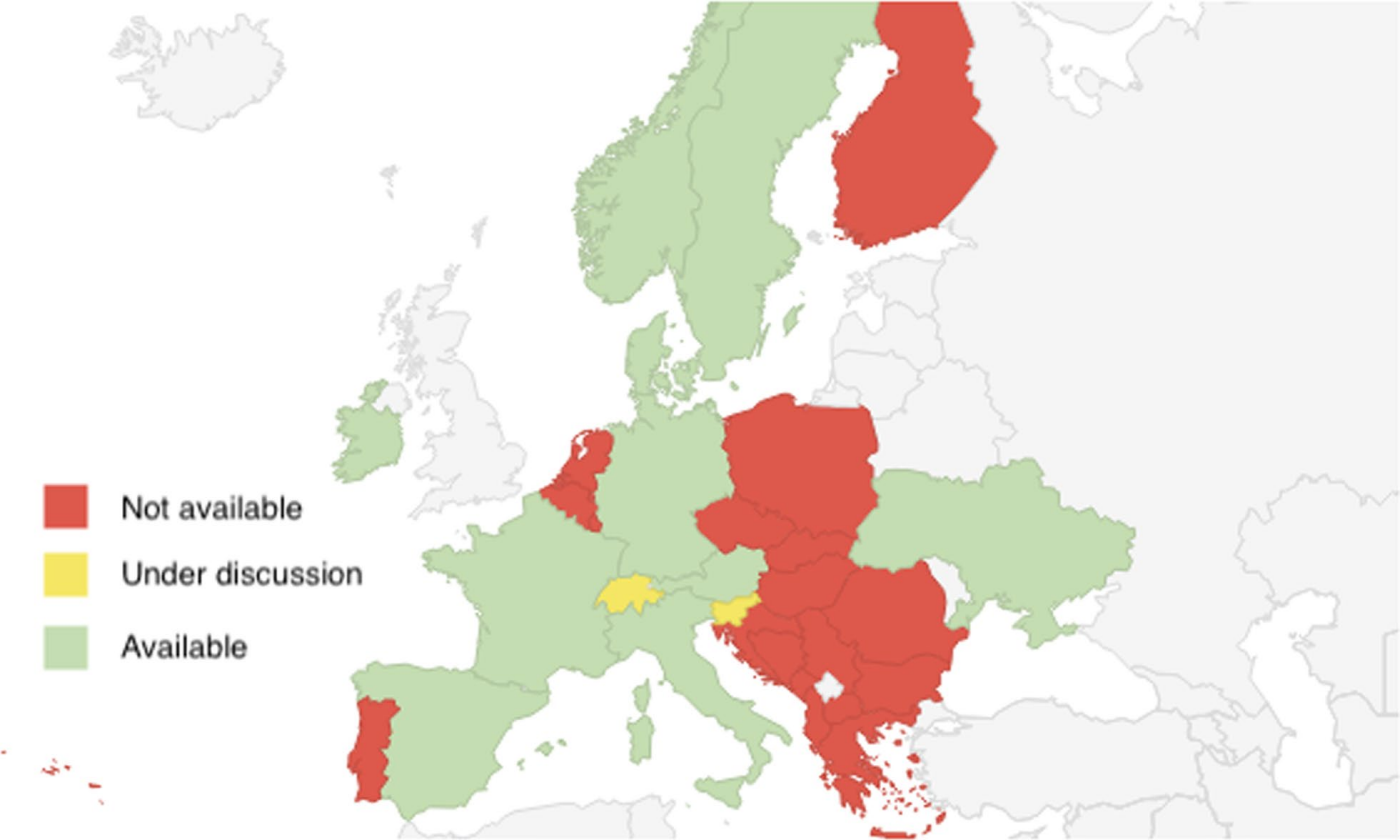
Availability of take-home naloxone

In C-EHRN monitoring, 12 European countries reported having THN programmes, a result that corresponded to numbers registered by the EMCDDA^2^ among its 30 member countries [21]. Also, Slovenia and Switzerland^3^ reported plans to make THN available soon (see Fig. 2).

However, even in countries where THN is officially available, C-EHRN monitoring showed that real access is still a challenge. Only FPs from four on the 12 countries (Georgia, Italy, Norway and Spain) affirmed that THN is widely accessible. A problem reported from some THN programmes was that they remained local and project-based and have not been expanded into mainstream practice in health services.

We have provided naloxone in Denmark based in a project setting for nine years. There is reluctance at the state level to give THN for all communities. This might change from 2020. (Denmark FP).

Another problem is the need for a medical prescription to acquire naloxone. In some countries, people who are opioid-dependent and enrolled in an OST programme are the only ones who can directly obtain the medication. Subsequently, as many active users do not have contact with a doctor, they are excluded from the possession of naloxone. Furthermore, people who are likely to witness an overdose (such as family or friends of people who use opioids) are not allowed to carry or administer naloxone.

In countries with TNH where a prescription is not needed (such as France, Italy and Ukraine), a problem encountered is the lack of widespread availability of naloxone. Moral judgement towards people who use drugs might be playing a role on that:

Pharmacies can sell naloxone without prescription due to advocacy by CSOs, especially the PWUD community. However, most pharmacies do not order naloxone, so for PWUD, it is problematic to get it easily. (Ukraine FP).

Only injectable naloxone is available in pharmacies […] but in reality many pharmacies do not have any stock of naloxone. Some of them still think it is only aimed at drug users and don't want them to be part of their clients. (France FP).

Finally, some THN programmes have unnecessary barriers in place [22] which are not compatible with a low threshold service:

Among the 138 local authorities that provided take-home naloxone in England in 2016/17. 18%  (25 local authorities) requires a person to be referred to a take-home naloxone provider. 17% (24 authorities) require a person to book and attend an appointment with a take-home naloxone provider, meaning that this is not available to someone that drops into the service provider without an appointment. And 20% (28 authorities) require a person to be assessed by a take-home naloxone provider. (UK FP).

In a few cases, even with the legal restrictions, naloxone distribution occurs informally (in an ad hoc and at a local level). People in need can then find ways to get access to the lifesaving medicine.

In Sweden, it is against the law to give a medication against a person’s will if you do not have medical training. Interpretation of the law is that if you are unconscious, you cannot give consent. This theoretically makes it impossible to provide naloxone to someone in need. However, we are finding ways to get around this barrier. You can delegate a person to administer naloxone to you, for example, a partner or wife/husband, but you both have to go to a doctor and pass the naloxone training course. (Sweden FP).

The civil society-led monitoring, thus, allowed to denounce the discrepancy between the officially reported availability of THN programmes and the actual access granted to people in need. Such detailed data are most valuable to inform OD prevention policies and guidelines for the implementation of THN programmes. Sufficient and good-quality information based on CSOs experiences is needed to feed into policy planning and systematically implemented actions. Only when data reflect what happens on the ground can one successfully push for the implementation of evidence-based policies to prevent overdoses.

## Identifying gaps in current data collection

Another vital contribution of CSO-led harm reduction monitoring was to identify gaps in current data collection carried out by other agencies in Europe. C-EHRN FPs were asked to analyse challenges and weaknesses regarding the official and available channels of information on drug-related overdoses in their country and which they used to gather information for their programmatic planning. Virtually, all FPs collect data through official sources, usually from governmental bodies: National (Public) Health Institutes, Forensic Institutes, National Statistics Institutes, Narcotics Agencies, other specific drug-related reports and EMCDDA National Focal Points.

C-EHRN FPS identified the following challenges and gaps around drug-related overdose data:

- Further forensic investigations are not always carried out if a different cause of death is determined. Toxicological analysis of death is often not performed due to financial costs

Further forensic investigations are not always carried out. For example, cardiac arrest may be identified as a cause of death, but drugs may not be sought in the blood, although, for example, cocaine overdose was not excluded as a causal cause of cardiac arrest. (Switzerland FP).

- Besides prescription drugs sold on the illicit market, personally prescribed pharmaceuticals can also be linked to drug-related deaths.
- Official data may lack validity, as many states/locations do not perform autopsies. Local harm reduction providers get, from their practice, higher numbers of drug-related deaths than those given by the government
- Data might also be unreliable in detecting the actual pattern of drug-related overdoses since, due to stigma, overdose deaths may be reported as having a different cause.

Because of stigma, many parents bribe the doctors to report the cause of death as something different than an overdose. (Greece FP)

- In European reporting, there is an unavoidable time lag of about two years. Data on drug-related deaths are first compiled at country-level in official national databases which takes a minimum of 12 months, before they can be accessed for reporting, analysis and synthesis at European level. This means that the information is too outdated to provide a timely policy response at the national or local level. Responses to sudden peaks in overdose could be improved if local and national stakeholders worked together to carry out their analyses, and enrich the findings by contextual data on the individual cases that might be accessible to civil society organisations.
- Finally, sudden changes in the way of collecting data can cause misleading conclusions about a country’s situation and response to OD.

Until 2016, suicide involving drugs was only registered as suicide in the Dutch national statistics. In 2016, the form of registration changed to register suicide involving drugs as drug-related deaths. Since then, data shows an increase in OD rates in the Netherlands, and so this rise might have a simple statistical explanation. (Netherlands FP).

Trying to fill the gaps left by official data collection, at least half of the C-EHRN FPs reported gathering complementary data on drug-related deaths through informal channels. Such data collection, however, is ad hoc and non-systematic. In most cases, the information comes from the clients of harm reduction programmes and staff from other harm reduction and drug treatment programmes. Harm reduction CSOs also collect information from medical staff, first responders (such as ambulance staff) or from local informants. The C-EHRN monitoring revealed that some CSOs are collecting their data in order to gain a better overview of their local or national context. In Czechia, for instance, the National Focal Point *SANANIM* partnered with another CSO to collect and analyse contextual data about deaths of their clients [23]. In the Netherlands, *Mainline* has produced a critical report about the official data on drug-related deaths [24] and has partnered with *the Trimbos Institute* and *De Regenboog* to record the number of drug overdoses in DCR’s in 2018, including non-fatal overdoses [25]. Another potential added value of CSOs could be to collect data on the context of overdose deaths. This could be, for instance, to collect anonymised qualitative data on the circumstances of overdose as known by service users as where and with whom PWUD were when they overdosed, if they had (regular) access to overdose prevention information and care, or what moral/legal grounds may be at play when deciding (not to) call for help or report an overdose.

## Complementing data from official agencies

Civil society-led monitoring of Harm Reduction can bring essential added value in terms of systematically complementing data reported by other monitoring agencies. Such data complementarity can play an essential role for optimising local planning (type and scale) of drug service provision and development of effective and respectful drug policies at the national level.

Having a systematic overview of current policies addressing OD prevention at the national level in the different European countries is crucial for strategic advocacy, planning and programming. The EMCDDA collected data on OD prevention policies in previous years, and the information has been used in EU Action plan reports. After 2013, however, in the context of a reform of its data collection tools, the agency stopped gathering such data. The C-EHRN monitoring resumed data collection on this issue by asking participants whether drug-related overdose deaths and ways to prevent them are mentioned in the respective national drugs strategy or action plan**.** Twenty out of 34 FPs reported having OD prevention mentioned in national policies. The civil society-led monitoring also found that 10 out of 34 FPs reported having nationally defined protocols for overdose management**.** These protocols provide, for instance, instructions for ambulance staff and other first responders; determine the right of police (not) to accompany an ambulance; provide guidelines on how to identify an OD; when, and to whom, to administer naloxone. Fewer FPs (from Italy, Luxembourg, Norway, Spain, and Sweden) reported having separate drug overdose prevention strategies or action plans, and an expert group is currently working to build one in France. In at least eight countries (Bosnia and Herzegovina, Finland, Germany, Montenegro, Portugal, Poland, Russia and Slovenia), FPs reported that drug-related deaths are mentioned neither in the national drug policy and federal guidelines nor in separate strategies or national protocols on OD. Perhaps even more importantly, the 2020 version of the monitoring also collects data on desired changes in current policies and how still inexistent policies should look like. Such information is crucial for advocacy and policy makers willing to implement changes.

The C-EHRN monitoring also gathered complementary data on OD prevention measures related to OD prevention and education to PWUD and their networks and on first responders. Most participants (27 out of 34 FPs) reported having OD prevention education and training for PWUD, their friends or family members. OD prevention education mostly comprised of delivering brochures, handbooks or online information, although training and information sessions for PWUD were also reported. Despite the high number of FPs indicating the existence of OD prevention education to this population, in all cases, CSOs were primarily responsible for delivering OD prevention.

PWUD only receive information on or participate in education/training about, overdose prevention measures when CSOs or outreach teams provide this kind of training. There is nothing formal. (Portugal FP).

This may imply a non-continuous and non-systematic service offer, which can compromise the OD prevention strategy. The information, thus, points at the need for advocacy with national and local governments to take up OD prevention strategies and assure systematic and continuous interventions.

**Fig. 3:**
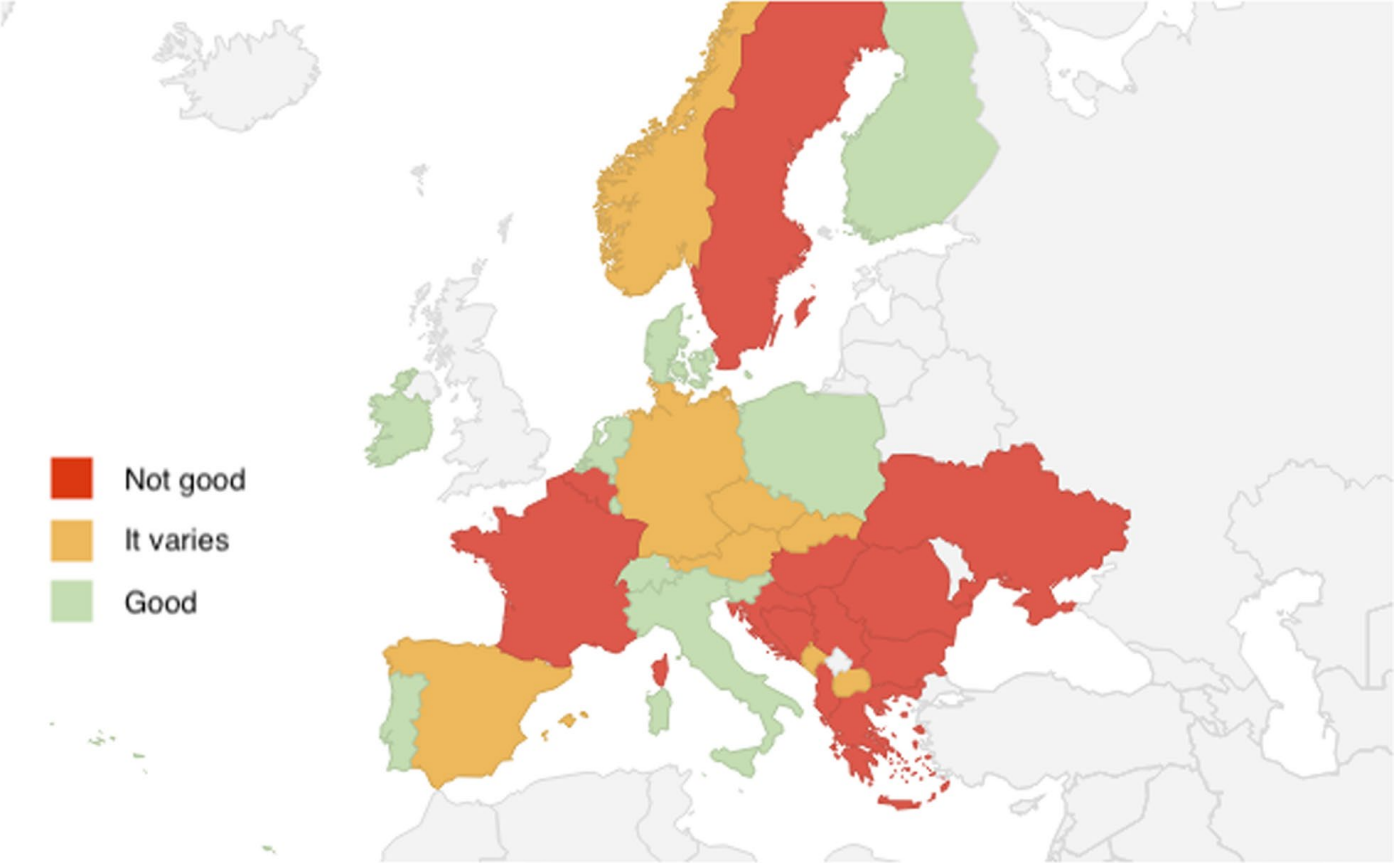
Evaluation of first responders to OD situations

C-EHRN FPs were asked to evaluate the training and capabilities of first responders (ambulance, fire brigade, police) for handling overdose situations in their country, region or city. Data gathered revealed a wide variation in responses (see Fig. 3). About a third of respondents (11 out of 34) considered first responder training and capabilities for handling overdose situations as good. Such evaluations were based on good performance and speed of first responders; good knowledge about OD; being equipped with naloxone; and having good collaboration with harm reduction services. Another important feature is for first responders to not report to the police, so PWUD and their networks do not feel threatened to call an ambulance.

Another third [10] of respondents mentioned that the readiness of first responders differs considerably. Several reported that ambulance crews do a good job, but other first responders (such as police and the fire brigade) are not prepared. Another problem lies in the prejudicial attitudes towards PWUD.

“Most of the services providers are trained to treat an overdose, but we found that there are many prejudices against the group of people in active drug consumption.” (Spain FP)

The final third of respondents [11] who considered the preparedness of first responders as not good based their evaluation on the absence of naloxone during first response; limited knowledge and training about OD; stigma and prejudice towards PWUD; and calling the police when there is an OD case.

We have been fighting for the emergency services not to call the police to OD cases - unfortunately with not so much success. We have several instances when police are called automatically. (Hungary FP).

Such information is essential to inform strategic CSO advocacy as well as policy planning.

## The relevance of the monitoring to FPs and CSOs

Finally, civil society-led monitoring should not only be relevant for national and European harm reduction advocacy activities but first and foremost for the work carried out by the civil society organisations themselves. A survey among all C-EHRN members revealed the significant potential that monitoring can have for advocacy purposes. Seventy-five per cent of the members indicated that a monitoring report could have a positive influence on their engagement in advocacy, and 85% that such a report can create opportunities for cooperation on national and European level.

During the process evaluation with FPs in October 2019 (mentioned in Fig. 1), most FPs (94%) answered that the monitoring results are useful for their work. Nevertheless, they suggested that the information needs to be tailored to facilitate local or national advocacy activities. This includes, for instance, having an executive summary of the report for advocacy purposes, possibly translated into different languages, as well as policy briefs and fact sheets focused on specific themes that need more attention. Moreover, CSOs recommended to produce social as well as traditional media messages, with infographics and easily sharable links for social media, and to use awareness day campaigns (such as OD prevention day) to release information. Recommendations are being taken on board in the second round of the C-EHRN monitoring, to be published in early 2021.

Along the process of the monitoring and during the evaluation, new relevant themes were suggested to be covered in the civil society-led monitoring of Harm Reduction in Europe. In the 2020 version, new sections were included on essential harm reduction services and the harm reduction response to the COVID-19 pandemic. A parallel short questionnaire will be sent to PWUD, to better represent the voices of the community on the ground. These variations show that the C-EHRN monitoring framework and questionnaires are not static and that constant evolution of civil society-led monitoring is needed to define and select the best indicators to fulfil its complementary role, reflecting in a timely manner the variations and constant changes present in the field of drug use and harm reduction. A challenge for civil society-led monitoring of Harm Reduction is to be able to respond quickly and flexibly adjust to new needs in the field, while maintaining a systematic approach, transparency and relevance to inform policymaking better.

## Conclusions

Monitoring of the quality and coverage of harm reduction services are of outstanding quality in Europe, above all due to the work of the EMCDDA. There exists an inherent need, however, to continuously further develop the indicators and analytical methods and to improve the transfer of data in order to timely inform and improve policy and practice in that field. As our experiences show, CSOs can play a crucial role in that effort. CSOs working for and with drug users play a vital role in developing and implementing effective measures to address the negative consequences of drug use. They work directly for and with drug users and have a good insight into their daily problems. This applies in particular to harm reduction services, which are the first entrance and contact point for drug users, due to their low-threshold approach. Due to their closer contact with important actors in the field, CSOs have access to timely and quality information with fundamental value to develop policies and practices in the area. Pre-condition is—as we learnt—that the scope of the questions is close to the unique experiences of respondents and that the guidelines and instructions are detailed and precise.

In this context, the C-EHRN monitoring framework is producing a rich and unique corpus of data which adds value to and complements the data reported in by other monitoring agencies. The data show a wide range of barriers and mismatches between the official situation (policies, strategies, guidelines) and the reality of harm reduction service providers and service users. They also document innovation, progress and a multitude of new opportunities for the harm reduction sector, which can inspire new governmental policies and CSOs advocacy strategies. Furthermore, C-EHRN monitoring identified critical gaps in current data gathering, opening the space to reformulate methods and indicators to better respond to the needs of CSOs working in the field, and reinforcing the role of CSOs in defining data needs for harm reduction policy and practice.

It is an ongoing process to address data quality issues and the sustainability of reporting adequately, and maintaining a long-term perspective is necessary. Nonetheless, civil society monitoring can and should continue to provide added value for monitoring the achievement of European and global targets. They can also enhance their capacity to interact with policymakers to reduce drug-related harms effectively.

## Supplementary information

**Additional file 1**. List of C-EHRN Focal Points.

**Additional file 2**. C-EHRN monitoring questionnaire 2019.

**Additional file 3**. C-EHRN monitoring questionnaire 2020.

## Data Availability

The monitoring questionnaires used in 2019 and 2020 are publicly available at https://www.correlation-net.org/monitoring/. The datasets used and analysed during the monitoring study are available from the corresponding author on reasonable request.

## Abbreviations

C-EHRN: Correlation-European Harm Reduction Network
CSOs: Civil society organisations
DAA: Direct-acting antiviral
DCR: Drug consumption rooms
EMCDDA: European Monitoring Centre for Drugs and Drug Addiction
EU: European Union
FPs: Focal Points
HCV: Hepatitis C virus
HIV: Human immunodeficiency virus
HRI: Harm reduction international
NPS: New psychoactive stimulants
NSP: Needle syringe exchange
OD: Overdose
OST: Opiate substitution therapy
PWUD: People who use drugs
SEG: Scientific expert group
TB: Tuberculosis
THN: Take-home naloxone

## Footnotes

1 In this article, *civil society* is used as an umbrella term that can include community-based organisations, non-government organisations, faith-based organisations, charities and voluntary organisations; CSOs perform various functions including service delivery, monitoring government behaviour, and advocacy on behalf of particular communities including marginalised groups [26].

2 At the time of questionnaire design, in the beginning of 2019, we were informed by the EMCDDA that they were remarkably expanding their data collection of THN programmes but as it was anticipated their reporting would take time we decided to include rather extensive THN question pattern on C-EHRN questionnaire. Due to increased EMCDDA’s data on THN programmes, however, in 2020 C-EHRN monitoring questionnaire there are now a) significantly less questions about THN, and these questions b) address the situations on the city level instead of country level.

3 Cyprus is also planning to include THN programmes. C-EHRN did not have a FP in the country during the 2019 round.
