# Supplementary material for: Harm reduction in Europe: a framework for civil society-led monitoring
Source: Harm Reduct J. 2021 Jan 6;18:3. doi: 10.1186/s12954-020-00451-7 (PMC7787243; doi:10.1186/s12954-020-00451-7)
Supplement: Supplementary file 1 — Additional file 1. List of C-EHRN Focal Points. [file 12954_2020_451_MOESM1_ESM.docx]

*Additional file 1: List of C-EHRN Focal Points*

Aksion Plus (**Albania**)

Suchthilfe Wien (**Austria)**

Free Clinic **(Belgium)**

PROI **(Bosnia and Herzegovina)**

Initiative for Health (**Bulgaria)**

Udruga Vida *(***Croatia)**

Sananim (**Czechia)**

Health Team for Homeless (**Denmark)**

A-klinikkasäätiö **(Finland)**

Federation Addiction (**France)**

Georgian Harm Reduction (**Georgia)**

DAH **(Germany)**

Positive Voice **(Greece)**

Rights Reporter Foundation **(Hungary)**

Ana Liffey, (**Ireland)**

Forum Droghe & Lila (**Italy)**

Dialogs (**Latvia)**

Coalition “I can live” (**Lithuania**)

JDH (**Luxembourg)**

Juventas (**Montenegro)**

HOPS – Healthy Options (**North Macedonia)**

Mainline (**Netherlands)**

Prolar **(Norway)**

Monar (**Poland)**

Apdes (**Portugal)**

Carusel **(Romania)**

AFEW **(Russian Federation)**

Scottish Drug Forum (**Scotland)**

Association Prevent (**Serbia)**

Odyseus (**Slovakia)**

Stigma **(Slovenia)**

Creu Roja (**Spain)**

Brukarföreningen Stockholm (**Sweden)**

**I**nfodrog **(Switzerland)**

Release (**United Kingdom)**

AFEW-Ukraine **(Ukraine)**
