# Supplementary material for: Harm reduction in Europe: a framework for civil society-led monitoring
Source: Harm Reduct J. 2021 Jan 6;18:3. doi: 10.1186/s12954-020-00451-7 (PMC7787243; doi:10.1186/s12954-020-00451-7)
Supplement: Supplementary file 2 — Additional file 2. C-EHRN monitoring questionnaire 2019. [file 12954_2020_451_MOESM2_ESM.pdf]

Correlation
European
Harm Reduction
Network

monitoring tool

Monitoring Harm Reduction Services
Version 1.0

Correlation Network
c/o Foundation De REGENBOOG GROEP
Droogbak 1d
1013 GE Amsterdam
The Netherlands

www.correlation-net.org

Correlation
European
Harm Reduction
Network

Correlation
European
Harm Reduction
Network

Index

Introduction 4

General Information 7

Hepatitis C 10

Overdose Prrevention 19

New Drugs & New Patterns of Consumption 33

Civil Society Involvement 44

Guidelines for answering

The following questionnaire consists of five parts.

The first part collects background information about your organization, and the experts and other organizations that provided with you information.

The second, third and fourth part, are the main sections of this survey. They contain questions about the responses to HCV [part two], Overdoses [part three] and new drugs and new patterns of consumption [part four] in your country.

The fifth part presents questions about civil society involvement in drug policy and related decision-making processes in your country.

How to collect information and answer the questionnaire?

Please first use the attached Word document to collect answers to all questions from your national experts (including yourself of course). Later on, as the dead line approaches, we will send you a link to the online version of the questionnaire, and ask you to move your answers there from the Word file. That means that we only receive ONE synthesized answer per country.

We suggest the following options for the data collection:

1. Answer yourself all questions on which you do have own expertise or where you are able to collect information by yourself. However, don't collect data only from official resources but from the viewpoint of civil society.

2. Send the questionnaire to other experts in your country, who have the relevant expertise to answer and mark the questions you want to have their answers on or copy/paste questions in a new document.

3. Another possibility is to place the questionnaire (word file) to a Dropbox, GoogleDrive or other such shared cloud service, and to ask your national experts to write their answers directly to a shared file.

4. You could also gather a group/network of other experts that can answer the questions.

» You can organize either a face-to-face meeting or e-meeting to discuss the questionnaire, and make a division of work among your experts (=to decide upon who will take the responsibility to answer different questions).

Please, remember that it is you who gathers all answers together and transfer them online (link to that will be sent to you later) to the Correlation Network Office.

Please also have in mind that it is not the purpose to prepare a representative data collection but rather an assessment of harm reduction experts in the three areas about the actual situation and the developments. You might also collect contradictory information or certain information may not apply for the entire country. Please describe that situation shorty in one of the open boxes.

Content is more important than style

» Do not worry too much about the language: your answers do not need to be linguistically groomed – the content is the more important than the style!

» Please provide us with any additional information you feel – even if it was not asked separately – is important to understand the situation with regard to harm reduction policy within your country. You can provide links, references, figures, tables etc. wherever relevant.

» Please feel also free to comment on the explanations for the current situation or changes in it. These comments can be subjective and anecdotal by nature – we are interested in your own interpretations why the state of affairs is like it is!

Feedback

Once you submit your questionnaire, we would like to ask about your experiences by answering our questions. However, please feel free to add any comments to the end of your answers.

Should you have any questions, please send an e-mail to:

Tuukka Tammi, tuukka.tammi@thl.fi
Eberhard Schatz, eschatz@correlation-net.org
Roberto Perez Gayo, rpgayo@correlation-net.org *(technical questions online survey)*

I - Background Information

General Information

1. Organization Details

Name	
Department	
Website	
Address	
Postal Code	
City	
Country	

2. Contact Person Details

First Name	
Family Name	
Role/Position	
Email Address	
Phone Number	

3. Other contributing organisations and their contact persons (please name at least one for each theme):

Hepatitis C

Overdose Prevention

New Drugs & New Patterns of Consumption

Civil Society Involvement

II - Hepatitis C

Changes in National Legislation

With the following questions, we want to assess the impact of national strategies or guidelines on the accessibility to testing and treatment for people who use injectable drugs from the viewpoint of services, working with people who use drugs.

EMCDDA collected Hepatitis C (HCV) country related information, please check: http://www.emcdda.europa.eu/publications/topic-overviews/hepatitis-policy_en#panel2

1. Is the EMCDDA information in the summary of your country up to date, or did anything changed recently in regard to new or updated hepatitis C strategies, guidelines etc.?

□ I don't know
□ The information given is still valid
□ The information needs to be updated. Please, specify: ____________________

____

____

2. What is the most relevant medical guideline in your country when it comes to HCV treatment?

□ No guidelines exist
□ I don't know
□ National guidelines
□ EASL guidelines

3. Name / link to the guidelines

____

____

____

4. In case you have any kind of HCV treatment guidelines, do these guidelines include specific measures for people who use drugs?

- ☐ Yes
- ☐ No

5. Do you think these guidelines impact accessibility to testing and treatment of people who use injectable drugs in your country?

- ☐ Yes
- ☐ No

6. Do these guidelines have impact on better access to the service provided by your organization?

- ☐ Yes
- ☐ No

7. Is there any important issue missing in those guidelines, do you have other comments on the guideline and its implementation?

With the following questions, we want to assess if there are still barriers for testing and treatment of people who use injectable drugs in your country:

8. Are there new drugs for treatment of hepatitis C (direct acting antivirals, DAAs) available in your country?

□ Yes
□ No

9. Are there new drugs for treatment of hepatitis C (direct acting antivirals, DAAs) accessible in your country?

□ Yes, with no restrictions
□ Yes, with restrictions
□ No

10. In case there are accessible, is there an official policy on the restriction for the use of new drugs for HCV?

□ Yes
□ No

11. In case there is an official policy restriction for the use of new drugs regarding the stage of liver disease, are they to be used only in patients with:

□ Cirrhosis (fibrosis stage 4)
□ Fibrosis stages 3 and 4
□ Fibrosis stages 1, 2, 3 and 4

12. Are the new drugs for hepatitis C allowed to be used for people who use injectable drugs in your country (accordingly to the guidelines)?

□ Yes
□ No

13. In case they are allowed to be used for people who use injectable drugs, are they applicable to:

- ☐ People who use injectable drugs on opiate substitution treatment
- ☐ Active people who use injectable drugs
- ☐ Former people who use injectable drugs

14. In practice, are the DAAs used according to the official policy?

- ☐ Yes
- ☐ No

15. In case they are not, what are the main differences / points of divergence?

16. Is treatment with the new drugs for hepatitis C reimbursed in your country?

- ☐ Yes - Health insurance
- ☐ Yes - Public health service
- ☐ Yes - Other, namely ______________________________

____

- ☐ No

17. Do you have any other comment on HCV policy or restrictions in practice? If yes, please describe.

Changes in the Continuum of Care

A good functioning of the continuum of care, including low threshold and harm reduction services, is increasingly important for the accessibility and the impact of HCV testing and treatment. With the following questions, we want to know, how the continuum of care is functioning in your country.

18. Where PWIDs can be tested for hepatitis C for HCV antibodies (oral swab or antibodies fingerprick)?

- □ At Gastroenterology Clinics
- □ At Infectious Disease Clinics
- □ At Centers for Treatment of Drug Addiction
- □ At Harm Reduction Services or Community Centers
- □ At General practitioner
- □ At pharmacies
- □ At prison
- □ Other, namely ______________________________

____

____

19. Where PWIDs can be tested for hepatitis C RNA (blood test)?

- □ At Gastroenterology Clinics
- □ At Infectious Disease Clinics
- □ At Centers for Treatment of Drug Addiction
- □ At Harm Reduction Services or Community Centers
- □ At General practitioner
- □ At pharmacies
- □ At prison
- □ Other, namely ______________________________

____

____

____

20. In case hepatitis C treatment is accessible for people who use drugs in your country, where are they treated for hepatitis C?

□ At Gastroenterology Clinics
□ At Infectious Disease Clinics
□ At Centers for Treatment of Drug Addiction
□ At General practitioner
□ At pharmacists
□ In prisons
□ Other, namely ____

____

____

21. Is there a clear linkage-to-care protocol/guidelines so that people diagnosed with HCV are referred directly to care management?

□ Yes
□ No
□ Don't know

22. Does your government monitor the numbers / proportions of people who progress through each stage of the HCV cascade of care?

□ Yes, at national level
□ Yes, at regional level
□ Yes, at local level
□ No
□ Don't know

23. Compared to the previous year, in the last year, service providers have invested attention to:

□ HCV Awareness Campaigns → □ more □ the same □ less
□ Testing on own location → □ more □ the same □ less
□ Treatment on own location → □ more □ the same □ less
□ Don't know

24. Compared to the previous year, did the coordination between health care providers on the one side (GPs, clinics) and social service providers (like NGOs, HR services) regarding HCV change?

- ☐ Information sharing → ☐ improved ☐ worsen ☐ remained the same
- ☐ Communication → ☐ improved ☐ worsen ☐ remained the same
- ☐ Service Provision → ☐ improved ☐ worsen ☐ remained the same
- ☐ Don't know

25. If in your country drug user groups (NGOs) exist, are they active for (political) awareness in regard of HCV?

☐ Yes, namely: ____

____

☐ Not to my knowledge

26. In your opinion, what are the main limitations for the harm reduction organisations in addressing HCV in your country, if any?

27. Other comments:

Correlation
European
Harm Reduction
Network

Would you like to add any other information or comment on HCV/OD/NDT in your country?

III - Overdose Prevention

General Policy Level

1. Are drug-related overdose deaths and ways to prevent them mentioned in your national drugs strategy or action plan?

□ Yes
□ No
□ Don't know

2. Is there a separate drug overdose (OD) prevention strategy or action plan in your country, region or city? If yes, please describe it shortly (and provide links if available).

□ Yes
□ No

Brief Description
(also distinguish between strategies/discussions at different levels):

Web link: ____

____

3. Are there nationally defined protocols for OD management? (For instance: How to identify an OD? When and who to administer Naloxone? What other processes needs to be considered? Think of ambulances and other first responders, what kind of conversations, right for police not to come with the ambulance, etc.)

□ Yes
□ No
□ Don't know

4. How do you get to know about the drug-related overdoses in your country? Please comment both official data on drug-related deaths and other, possibly your own information channels.

Drug-related Deaths

Other, possibly your own information channels

5. Please take a look at your Country Profile (2018) at the EMCDDA website, (http://www.emcdda.europa.eu/countries_en) and the sections on "Drug-induced deaths and mortality" [under 'Drug Harms')]. Have you provided data to your national EMCDDA focal point for this report? If yes, what data?

☐ Yes
☐ No

I contributed with the following data:

6. Do you find the information at this section of your country report up to date and sufficient?

- ☐ Yes
- ☐ No

7. Do you have other comments on the country report?

Naloxone

8. Is take-home-naloxone available in your country?

- ☐ Yes
- ☐ No. Please describe the barriers/reasons

10. If take-home-naloxone is available, is it used as it should be?

- ☐ Yes
- ☐ No. Please describe the barriers in practice

11. If take-home-naloxone is available, who is in charge of THN programmes?

12. If take-home-naloxone is available, what is the legal status of naloxone?

- ☐ Only with private prescription (doctor)
- ☐ With prescription (paid by health insurance)
- ☐ Over the counter status (without prescription in pharmacies)
- ☐ By harm reduction providers without prescription

Comments:

13. What is the cost of a single dose (ampoule/spray)?

14. Are there any variations in prices?

15. For whom (which groups of people) is naloxone accessible?

16. What kind of naloxone is available (for iv/im use; for iv/im use but with nasal applicator; nasal spray).

17. Is it training mandatory for carrying naloxone?

- ☐ Yes
- ☐ No

18. If yes, who are the trainers and who is eligible as a trainee?

Trainers

Trainees

19. How many people got naloxone training in your organisation?

- ☐ ____
- ☐ I don't know
- ☐ None

20. How many people got naloxone training in your city?

- ☐ ______________________ (also tell during which period if you know)
- ☐ I don't know

21. How many people got naloxone training in your country?

- ☐ ______________________ (also tell during which period if you know)
- ☐ I don't know

22. Is naloxone available in prison?

- ☐ Yes
- ☐ Yes, but only in some prisons. Please indicate number: ______________
- ☐ No
- ☐ I don't know.

23. If yes, which groups can use naloxone:

- ☐ Inmates
- ☐ Staff
- ☐ Medical staff
- ☐ I don't know

24. Do you have pre-release naloxone programmes ?

- ☐ Yes
- ☐ Yes, in some prisons. Please indicate number.
- ☐ No
- ☐ I don't know

25. In your country, is there a strategic plan for increasing access to nalaxone?

- ☐ Yes
- ☐ No

26. If you do, who made the plan:

- ☐ Government
- ☐ NGO. Please indicate which one(s)

- ☐ Other. Please indicate who.

27. If you do not have THN programmes, are there any current professional or political initiatives or discussions to start these programs or widen the use of naloxone?

- ☐ Yes
- ☐ No

28. If yes, please describe these initiatives/discussions shortly (who, when, what, why)

Drug Consumption Rooms

There will be a separate data collection on drug consumption rooms (DCRs) so only the following two questions here:

29. Do you know of any - professional or political - new initiatives to start new DCRs in your country?

☐ Yes
☐ No

Please, describe briefly.

30. Is there a legal framework which allows DCR experimentation in your country?

Opioid Substitution Theraphy

Prison release is a risky period (48h) from the viewpoint of OD. That's why Information and education is needed for both inmates and prison staff (esp. health care). The same concerns in-patient treatment for people with opioid dependence. The WHO has estimated that 20% of DRDs appear in connection to prison release or treatment relapse.

31. Is OST available in prisons in your country

☐ Yes
☐ No

33. Is there national mandatory or other routine information provided on the increased risk of OD in in-patient drug-treatment services (in connection to quitting treatment in OST centres, or in connection to prison release)?

☐ Yes
☐ No
☐ I don't know

34. Are there OD prevention responses linked to the prison release in your country?

☐ Yes
☐ No

Please, describe briefly.

35. Is it OST systematically continued with referral from prison health care to a new treatment provider in the community?

- ☐ Yes
- ☐ No

Other OD Prevention Measures

36. How would you assess the first responders' (ambulance, fire brigade, police) training and capabilities for handling overdose situations in your country/region/city?

37. Is there information and/or education/training available for the PWUD (and their friends & family members) on overdose prevention measures?

- ☐ Yes
- ☐ No

Please provide a link and/or describe what kind of information/education.

38. Is there information and/or education available for different professionals on overdose prevention measures?

- ☐ Yes
- ☐ No

Correlation European Harm Reduction Network

Please provide a link and/or describe what kind of information/education.

39. Is there Information and/or campaigns for the general public on overdose prevention measures?

☐ Yes
☐ No

Please, describe shortly what kind of information / education measures.

40. Are there drug-checking services in your country targeting especially overdose prevention?

☐ Yes
☐ No

41. Are there fentanyl / other potent new synthetic opioids (SO) available?

☐ Yes
☐ No

42. Do you know of any recent changes in the situation in your country/region/city?

☐ Yes
☐ No

Please, describe briefly

43. Are there fentanyl / other potent new synthetic opioids (SO) information or campaigns (for users/others)?

- ☐ Yes
- ☐ No

44. Are there fentanyl test stripes being used in your country?

- ☐ Yes
- ☐ No

OTHER DRUGS THAN OPIOIDS

45. Are there ODs and related responses to other drugs than opioids (NPS, GHB, MDMA, cocaine, etc.)?

- ☐ Yes
- ☐ No
- ☐ I don't know

If there are any, please, describe briefly

Correl
European
Harm Red
Network

Would you like to add any other information or comment on HCV/OD/NDT in your country?

IV - New Drugs & New Patterns Of Consumption

PLEASE NOTE: As our monitoring activities target new trends in drug use and drug markets, the focus is primarily but not exclusively on so-called NPS, but could also include changes in use of traditional drugs or changes in route of administration of those drugs.

ND&NP

Please answer in order of estimated popularity according to your opinion, where 1= most popular, and 5 = least popular of these 5 most popular drugs (and feel free to add comments& additional information after the questions);

1. At the moment, what are the 5 predominant 'traditional' drugs of use in your city?

1.
2.
3.
4.
5.

□ I don't know

2. At the moment, what are the 5 predominant 'traditional' drugs of use in your region?
[Please answer only if the information is different from the city level]

1.
2.
3.
4.
5.

□ I don't know

3. At the moment, what are the 5 predominant 'traditional' drugs of use in your country?
[Please answer only if the information is different from the city/regional level]

1.
2.
3.
4.
5.

□ I don't know

Please answer in order of estimated popularity according to your opinion, where 1= most predominant, and 3 = least predominant of these 5 most new substances

4. At the moment, what are the 3 predominant new substances of use in your city?

1.
2.
3.

□ I don't know

5. At the moment, what are the 3 predominant new substances of use in your region?
[Please answer only if the information is different from the city level]

1.
2.
3.

□ I don't know

6. At the moment, what are the 3 predominant new substances of use in your country?
[Please answer only if the information is different from the city/regional level]

1.
2.
3.
□ I don't know

Please comment how these substances (related to questions 4–6) were noticed:

Developments in the Last 6 Months

7. During the last 6 months, have you noticed any new substance appearing in the local, regional or national market?

□ Yes
□ No

In case you have noticed any new substance(s), please, fill in the ND&NP Annex (I) as well. Otherwise, please, skip this section.

8. During the last 6 months, have you noticed any new group of people using substances?

□ Yes
□ No

In case you have noticed any new group(s) of people using substance(s), please, fill in the ND&NP Annex (II) as well. Otherwise, please, skip this section.

9. Did any other worth mentioning developments occur?

□ Significant changes in route of administration
□ Source of purchase (e.g. internet, …)
□ Change within specific groups
□ New combinations of different drugs
□ Changes in prices
□ Change in availability
□ Change Perceived quality of certain drugs
□ Other. Please, specify.

10. Were any of the above mentioned developments checked with other stakeholders?

□ Yes
□ No

If yes, please indicate name of the organisation / person:

Correlation
European
Harm Reduction
Network

11. Please, add relevant literature [including media resources] available.

ND&NP Annex (I)
New Substances

Please, copy, paste and fill this set of questions as many times as new substances have been identified.

1. Name(s) of drug as which it was sold/marketed:

2. When did it first appeared in the market?

3. Exact content [if lab analysed]:

4. The drugs appeared as:

- ☐ Drug of choice
- ☐ Adulterant of another substance

5. Where was the drug sold?

- ☐ Online
- ☐ Offline

Please, indicate [online: name web address, vendor; offline: name city/region]

6. Price per gram/tablet/blotter/capsule/liquid:

7. If used, desired effects?

8. If used, undesired effects?

9. Have you heard of any incidents after using this substance? Multiple choice possible

- ☐ Yes, non-fatal.
- ☐ Yes, fatal.
- ☐ No.

10. If used, in what scene: (e.g. type of festival, at home,…)

11. If used, what category of population: (e.g. homelessness, students, ….)

12. Appearance of the substance

- ☐ Powder
- ☐ Tablet with groove
- ☐ Table without groove
- ☐ Capsule
- ☐ Crystals
- ☐ Liquid
- ☐ Herbal mixture
- ☐ Other. Please, specify ______________________________

____

13. Colour of the substance

14. Other relevant information / specifics:

ND&NP Annex (II)
New Groups of People Using Substances

15. Please, describe the groups as accurately and sensitively as possible

16. What substance has been used?

17. In which way it has been used?

18. If know, why is this substance been used?

19. Other relevant information / specifics:

Correl
European
Harm Red
Network

Would you like to add any other information or comment on HCV/OD/NDT in your country?

V - Civil Society Involvement in Drug Policy

Civil Society Participation

1. Is there in your country any structural [more or less permanent] information and exchange mechanism between policy makers and civil society organizations in the field of drug policy?

□ Yes
□ No

2. If yes, which one of the following levels?

□ **Information**

This is a relatively low level of participation. It consist of a two-way mutual process between public authorities and CSOs of information provision and access to it.

□ **Consultation**

Through this and ad hoc mechanism through which public authorities ask CSOs for their opinion on a specific policy topic, or development.

□ **Dialogue**

Dialogue entails a two-way communication mechanism built on mutual interests and potentially shared objectives to ensure a regular exchange of views.

□ **Partnership**

This mechanism articulates shared responsibilities for each step of the policy making process: agenda setting, policy drafting and implementation of activities. As such, this structure of participation is the most comprehensive and it is based on co-management.

3. Is your organization involved in these discussions?

□ Yes
□ No

How or why not – please comment shortly your answer:

If not all, which are the main civil society organisations involved in these discussions:

4. In case you are involved, from 0 to 10, what evaluation would you give to this mechanisms of exchange?

Why? – please comment shortly your answer:

5. Is your organization in any sort of relationship with your national EMCDDA focal point?

☐ Yes
☐ No

6. In case you are, could you please describe this relationship briefly?

7. In case you are not, could you please describe the reasons?

8. In case you are not, would you be willing to contribute more to the national reporting to the EMCDDA?

☐ Yes
☐ No

Would you like to add any other information or comment on HCV/OD/NDT in your country?

Correlation
European
Harm Reduction
Network
