# Supplementary material for: Harm reduction in Europe: a framework for civil society-led monitoring
Source: Harm Reduct J. 2021 Jan 6;18:3. doi: 10.1186/s12954-020-00451-7 (PMC7787243; doi:10.1186/s12954-020-00451-7)
Supplement: Supplementary file 3 — Additional file 3. C-EHRN monitoring questionnaire 2020. [file 12954_2020_451_MOESM3_ESM.pdf]

CIVIL SOCIETY MONITORING OF HARM REDUCTION IN EUROPE 2020

Guidelines for collecting data and answering the questionnaire

The following questionnaire consists of seven parts and has a total of 83 questions:

1. Background information (2 questions)
2. Essential harm reduction services (6 questions)
3. Hepatitis C (20 questions)
4. Overdose prevention (23 questions)
5. New Drug Trends and synthetic opioids (7 + 9 = 16 questions)
6. Civil Society Involvement in drug policy (6 questions)
7. Harm reduction response to COVID-19 (10 questions)

You have received both a Word document and a link to an online survey. We suggest you use the Word document to collect answers from your national experts (and your answers). That is the easiest way to collect information and avoid technical problems. When the answers are complete, please transfer them to the online survey. You can also fill in the online survey directly. It is possible to answer it in parts if you do it from the same computer. Your answers will be automatically saved, and you can go back to them later (using the same link and the same device). **We ask you to send us only ONE synthesised answer, via the online survey link.**

Please provide answers from the viewpoint of civil society. You are not expected to know or to collect official data. We suggest that you answer all questions on which you do have the expertise or can collect information by yourself. For the other questions, you can contact other experts from the civil society who have the relevant expertise.

When asking for information from other experts in your country, you can use the signed introduction letter you have received. It explains about the Monitoring and your role as a C-EHRN Focal Point.

A few suggestions on how to collect information from experts:

- In the word document, mark the questions you want to have their answers on or copy/paste questions in a new document or e-mail
- You may also place the word file to a Dropbox, GoogleDrive or other such shared cloud service, and to ask your national experts to write their answers directly to a shared file.
- You could also gather a group/network of other experts that can answer the questions (for instance by organising an e-meeting).
- It might be that you collect contradictory information or certain information does not apply for the entire city or country. Please describe that shortly in one of the open boxes.

Content is more important than style. Do not worry too much about the language. Please do provide us with any additional information you feel is essential to understand the situation about harm reduction policy within your country. Also, feel free to tells us about your own experiences and interpretations on why the state of affairs is like it is!

Please, remember that it is you who gathers all answers and transfer them via the survey link to C-EHRN Office. Do not share the survey link with other experts.

Our team at C-EHRN is ready to support you. **Should you have any questions, please send an e-mail**

to monitoring@correlation-net.org In the half of June we will organise an **online Q&A group session** about the Monitoring. All FPs are invited to join and will receive an invitation with the date.

Abbreviations

C-EHRN – Correlation – European Harm Reduction Network

CS – Civil Society

CSOs – Civil Society Organizations

DAAs - Direct-Acting Antivirals

DCR – Drug Consumption Rooms

EMCDDA – European Monitoring Center for Drugs and Drug Addiction

EU – European Union

GPs – General Practitioners

HCV – Hepatitis C

HCV RNA –Hepatitis C virus, or ribonucleic acid (RNA)

HIV - Human immunodeficiency virus

LGBTQI – Lesbians, Gays, Bisexuals, Transgenders, Questioning (or Queer) and Intersex

NGO – Non-governmental Organisation

OD – Overdose

OST – Opioid Substitution Treatment

PWID - People who inject drugs

PWUD – People who use drugs

WHO – World Health Organization

1. Background information

1. Details

Your first name	
Your Family Name	
Your role/Position	
Organisation's name	
Organisation's City	
Organisation's Country	

2. Would you like to acknowledge any contributors for their help in answering this survey? If yes, please mention their names and organisations below:

2. Essential Harm reduction

This is a new section of the C-EHRN Monitoring. It aims to monitor developments on essential harm reduction services in your city in the last 12 months.

1. Are harm reduction services in your city able to provide services for the following populations?

	To a great extent	Somewhat	Very Little	Not at All	Not relevant to my city
People who inject opiates (including synthetic opioids)					
People who inject stimulants or new psychoactive substances					
People who smoke opiates					
People who smoke stimulants or new psychoactive substances					
People who use intranasal amphetamines/cocaine/ cathinone, etc.					
Sex workers					
People experiencing homelessness					
Women					
LGBTQI					
Young people who use drugs (under 18 years of age)					
EU- Migrants					
Non-EU migrants					
People in prison settings					

Other populations assisted or any further comments

2. Are the following services available in your city for people who use drugs?

	To a great extent	Somewhat	Very Little	Not at All	Not relevant to my city
Outreach work					
Peer support					
Needle Syringe Exchange					
Safer smoking kits					
Safer intranasal kits					

Drug Consumption Room					
Drop-in centre					
Drug Checking					
Fentanyl test strips					
OST					
OST in prison					
Naloxone					
Take-home naloxone					
Naloxone in prison					
Prevention of sexual risks					
HIV services (prevention, testing or treatment)					
Specific employment opportunities/ income generation for PWUD					
Online harm reduction					
Shelters					
Legal support					

Other relevant services or any further comments

3. Do you feel the current harm reduction services at your city can meet the needs of PWUD?
   ( ) Yes
   ( ) No

4. If not, what are the three major needs of PWUD in your city? Please explain.

5. What is needed to improve the harm reduction services in your city?

6. How does your city compare with the national situation in terms of harm reduction coverage?
   ( ) my city has better coverage
   ( ) my city has lower coverage
   ( ) the situation is similar
   ( ) I do not know
   Please give some details

3. Hepatitis C

The definitions of terminology used in this section:

- **HCV treatment**: treatment with the new drugs, direct-acting antivirals (DAAs)
- **"People who inject drugs" (PWID)** include all the subgroups, if not stated otherwise:
    - currently active injectors,
    - former/current injectors on opioid substitution treatment (OST)
    - occasionally injecting drugs,
    - no more injecting drugs,
    - former injectors: no more injecting drugs and not on OST,
    - ever injectors: all mentioned above.

Changes in National Legislation

With the following questions, we want to assess the impact of national strategies or guidelines on the accessibility to testing and treatment for people who use injectable drugs (PWID). We want the viewpoint of services working with people who use drugs.

Please check the EMCDDA collected Hepatitis C (HCV) country related information:
http://www.emcdda.europa.eu/publications/topic-overviews/hepatitis-policy_en#section4

1. Is the EMCDDA information in the summary of your country up to date, or did anything changed recently regarding new or updated hepatitis C strategies, guidelines etc.?

☐ I don't know

☐ The information given is still valid

☐ The information needs to be updated →Please specify below the updates needed and, if possible, provide a link to any updated document you have:

2. Which guidelines for HCV testing and treatment of people who inject drugs (PWID) are used in your country?

☐ I don't know → Please go to question 5

☐ No guidelines available → Please go to question 5

☐ EASL guidelines

☐ National guidelines with PWID included

☐ Separate national guidelines for PWID

☐ Other guidelines (i.e. WHO) → Please provide the name of the guideline below:

3. Do you think these guidelines impact accessibility to testing and HCV treatment of people who inject drugs (PWID) in your city?

☐ **Yes – positively** → Please check below how (check all that apply):

- ☐ better access to CSO services
- ☐ better access to specialised HCV services
- ☐ better access to HCV information and counselling
- ☐ better access to HCV testing
- ☐ better access to HCV treatment
- ☐ other - specify:

☐ **Yes – negatively** → Please check below how (check all that apply):

- ☐ HCV testing is not possible outside the healthcare system
- ☐ HCV treatment is not possible outside the specialised healthcare system
- ☐ HCV treatment is prescribed only by specialists
- ☐ other - specify:

☐ **No** → Is there a mismatch between the guidelines and real-life situations? Please explain:

4. Is there any vital issue missing in those guidelines? Do you have other comments on the guidelines and their implementation?

With the following questions, we want to assess if there are still barriers to testing and treatment of people who inject drugs (PWID) in your city:

5. Are the new drugs for the treatment of hepatitis C (direct-acting antivirals, DAAs) accessible in your city?

☐ **Not accessible**

☐ **Yes, with no restrictions**

☐ **Yes, with restrictions**→ Please answer below (check all that apply):

- ☐ <u>restrictions to fibrosis stage</u>
  - ☐ accessible only for F4 (cirrhosis)
  - ☐ accessible only for F3 and F4

☐ accessible only for F2, F3, F4

☐ accessible for F1, F2, F3, F4

☐ other – specify:

☐ restrictions to injecting drug use

☐ accessible for former injecting drug users

☐ accessible for drug users on opioid substitution treatment

☐ accessible for currently active drug injectors

☐ accessible for all ever injectors

☐ other – specify:

☐ other restrictions – specify:

6. In case the guidelines allow the use of direct-acting antivirals (DAAs) for people who inject drugs (PWID), are they applicable to:

☐ Persons on opioid substitution treatment (OST)

☐ Persons who currently actively inject drugs

☐ Former injecting drug users, no more injecting and not on OST

☐ Persons who ever injected drugs

☐ Not allowed for any subgroup of people who inject drugs

7. In practice, in your city, are the direct-acting antivirals (DAAs) used according to the official policy?

☐ Yes

☐ No ⟶ Please specify the main differences/points of divergence:

8. Is treatment with the new drugs for hepatitis C (DAAs) reimbursed in your city?

☐ Not reimbursed

☐ Yes - with no limitations

☐ Yes – with limitations ⟶ Please specify the limitations to reimbursement:

9. Do you have any other comment on the policy or the restrictions to HCV management in practice?

Changes in the Continuum-Of-Care

The good functioning of the continuum-of-care, including low threshold and harm reduction services, is increasingly essential for the accessibility and the impact of HCV testing and treatment. With the following questions, we want to know how the continuum-of-care is functioning in your city.

10. Where can PWID be tested for HCV using point-of-care quick antibody testing (detection of anti-HCV in oral swab or finger prick)?

☐ At Gastroenterology Clinics
☐ At Infectious Disease Clinics
☐ At Drug Treatment Clinics
☐ At Harm Reduction Services or Community Centers
☐ At General practitioners
☐ At pharmacies
☐ At prisons
☐ Self testing
☐ Other → Please specify

11. Where can PWID perform a confirmatory blood testing for HCV RNA?

☐ At Gastroenterology Clinics
☐ At Infectious Disease Clinics
☐ At Drug Treatment Centers
☐ At Harm Reduction Services or Community Centers
☐At General practitioners
☐ At pharmacies
☐ At prisons
☐ Other → Please specify

12. Where can HCV-infected PWID perform non-invasive diagnostic procedure for the evaluation of the stage of disease (i.e. Fibroscan®)?

☐ At Gastroenterology Clinics

☐ At Infectious Disease Clinics

☐ At Drug Treatment Centers

☐ At Harm Reduction Services or Community Centers

☐At General practitioners

☐ At prisons

☐ Other → Please specify

13. In case the direct acting antivirals (DAAs) are accessible for people who inject drugs (PWID), where are they treated for hepatitis C?

☐ At Gastroenterology Clinics

☐ At Infectious Disease Clinics

☐ At Drug Treatment Clinics

☐ At Harm Reduction Services or Community Centers

☐ At General practitioners

☐ At pharmacists

☐ In prisons

☐ Other → Please specify

14. Who can legally prescribe direct acting antivirals (DAAs)?

☐ Specialists of gastroenterology/hepatology

☐ Infectious Diseases specialists

☐ General practitioners

☐ Pharmacists

☐ Nurses

☐ Other → Please specify

15. Is linkage-to-care for people who inject drugs (PWID) achieved by a written protocol/guidelines? (think of an agreed protocol to refer clients e.g. from a HR service to other treatment and care).

☐ Yes
☐ No
☐ Not relevant
☐ I don't know

16. Compared to 2018, in 2019 have service providers for people who inject drugs (PWID) in your city invested attention to the following?

	Yes, it remained the same	Yes, and it was better	Yes, but it was worse	No, they didn't invest on it	I don't know
HCV Awareness Campaigns					
Testing on own location					
Treatment on own location					

17. Compared to 2018, in 2019, did the coordination between health care providers (GPs, clinics) and social service providers (like NGOs, HR services) regarding HCV change?

	No, it remained the same	Yes, and it was better	Yes, and it was worse	I don't know
Information sharing				
Communication				
Service Provision				

18. If organised drug user groups exist in your city, are they active for (political) awareness in regard to HCV?

☐ Not to my knowledge

☐ Yes,→ Please tell us more details:

19. Are there limitations for the harm reduction organisations in addressing HCV in your city?

☐ Not to my knowledge

☐ Yes, → Please tell us which limitations you see:

☐ The weakness of harm reduction services
☐ Lack of funding
☐ Lack of staff
☐ Lack of knowledge
☐ Lack of recognition

☐ Lack of political support

☐ Lack of integration with the healthcare system

☐ Other – please specify:

20. Would you like to add any other information or comment on the management of HCV in your city?

4. Overdose Prevention

This section is a follow-up on the questions we sent out last year and aims to monitor developments in the area of overdose prevention in the last 12 months. In this Monitoring, we consider both fatal and non-fatal overdose occurrences. We also consider overdoses caused by any illicit drug or licit drugs used outside a medical prescription scheme.

General questions

1. Do you know of any guidelines for overdose prevention in your city that apply to your work? Please check all that apply.
   ( ) There are national guidelines for overdose (OD) prevention
   ( ) There are regional guidelines for OD prevention
   ( ) There are local guidelines for OD prevention
   ( ) Specific interest groups and associations have guidelines
   ( ) OD prevention is mentioned in the national drug strategy
   ( ) There are no guidelines
   ( ) I do not know
   ( ) Others (please specify):

2. Are there any important issues missing in these guidelines, do you have other comments on the guidelines and their implementation? If there is no guideline at all, please tell us what an ideal guideline should in your opinion address/cover.

3. In the last year, have you heard of overdoses involving the following drugs in your city?

	**Frequently**	**Sometimes**	**Rarely**	**Never**	**I don't know**
**Heroin**					
**Fentanyl**					
**Other synthetic opioids**					
**Cocaine**					
**Base or crack cocaine**					
**Methamphetamine**					
**Others**					

Which other drugs or any other observations?

4. Could you provide some examples of typical characteristics of OD victims and the circumstances/locations of their deaths? Please specify the drug(s) when known.

5. Which are the main challenges regarding the overdose response in your city in the last year? Please give us some examples.

6. In the last year, have there been any overdose awareness campaigns in our city? Please check all that apply.
   ( ) yes, for overdose in general
   ( ) yes, for opioids
   ( ) yes, for stimulants
   ( ) yes, for synthetic opioids (fentanyl, etc.)
   ( ) yes, for New Psychoactive Substances
   ( ) there were no campaigns

7. Is overdose response training available in your city? Please check all that apply.
   ( ) yes, for people who use opioids
   ( ) yes, for people who use other drugs than opioids
   ( ) yes, for friends and family of PWUD
   ( ) yes, for harm reduction staff
   ( ) yes, for medical staff
   ( ) there is no training, but there is information available
   ( ) I don't know
   ( ) others

8. Have activities on overdose prevention in your city improved in the past year?
   ( ) Yes
   ( ) No
   ( ) I don't know

9. Which changes in overdose prevention activities would you like to see in your city?

10. How does your city compare with the national situation in terms of overdose prevention?
    ( ) my city offers better prevention
    ( ) my city offers lower prevention
    ( ) the situation is similar
    ( ) I do not know
    Please give some details

Opiate-specific questions

Naloxone

11. Is naloxone available in your city:
    ( ) yes
    ( ) No →Please go to question 16.
    ( ) I don't know →Please go to question 16.

12. If naloxone is available in your city, who has access to it? Please check all that may apply.
    ( ) medical staff at harm reduction services
    ( ) medical staff at hospitals
    ( ) medical staff at ambulances
    ( ) harm reduction providers
    ( ) PWUD
    ( ) family and friends of PWUD

13. If naloxone is available in your city, how does that happen? Please check all that may apply.

    ( ) People who use opioids have access to take-home naloxone through prescription
    ( ) Naloxone can be purchased without prescription in pharmacies
    ( ) Drug service providers can distribute naloxone
    ( ) Naloxone is reimbursed by health insurance
    ( ) for take-home
    ( ) Intranasal naloxone is available
    ( ) Injectable naloxone is available
    ( ) training is available for staff administration
    ( ) training is available for peer administration
    Observations

14. To your knowledge, is there a policy change in process to increase access to naloxone in your city?
( ) Yes
( ) No
( ) I don't know
Please comment:

15. Which were the main challenges in your city regarding access to naloxone in the last year? Please give us some examples.

16. Which changes would you like to see regarding naloxone availability in your city?

17. How does your city compare with the national situation in terms of naloxone availability?
( ) my city offers better coverage
( ) my city offers lower coverage
( ) the situation is similar
( ) I do not know
Please give some details

OST (Opioid Substitution Treatment)[1]

18. Which of the following OST are available in your city?
( ) Methadone
( ) Buprenorphine
( ) Medical heroin
( ) Morphine
( ) Others ____
( ) OST is not available

[1] Also called MAT "Medically Assisted Treatment"

19. What factors limit OST accessibility in your city? Please rate them according to the level of the barrier they represent to achieving enough OST coverage.

	**Not a problem**	**Slightly problematic**	**Moderately problematic**	**Problematic**	**Very problematic**
Lack of prescribers					
Legal restrictions on OST					
Age restrictions					
**U**rine drug testing requirements					
Waiting lists					
Limited hours					
Stigmatisation					
Requirements for documentation					
Requirements for social coverage or medical insurance					
A requirement that people abstain from illegal drugs					
A requirement that people participate in meetings					
Cost restrictions					
Inadequate supply at pharmacies					
Others					

Please give us some examples

20. Has access to OST in your city improved in the past year?
( ) Yes
( ) No
( ) I don't know
Please comment:

21. In your opinion, what should change regarding access to OST in your city?

22. How does access to OST your city compare with the national situation?
    ( ) my city offers better coverage
    ( ) my city offers lower coverage
    ( ) the situation is similar
    ( ) I do not know
    Please give some details

23. Would you like to add any other comment or information about overdose prevention in your city?

5. New Drug Trends

This section is a follow-up on the questions we sent out last year and aims to monitor developments in the area of drug use and its users in the last 12 months.

Was the data provided below collected by people who are in direct contact with your target group(s)?	Yes/No

Changes in drug use in your city

1. In the previous year, have you witnessed any new developments regarding the use of drugs in your city amongst your target group(s):
   a. The emergence of a **new or unknown substance**?

( ) No, no developments were witnessed among our target group(s) in our city. → Please go to question 1b.

( ) Yes. Please tell us:

Which group(s) of PWUD is/are using this substance?	
Name(s) of the substance?	
What is its supposed content?	
When did it appear for the first time on the market?	
In what form does it appear? (powder, pill etc.)	
What is the colour of the substance?	
How is this substance being used? (Snorting, smoking, injecting, etc)	
Why is this substance used? (e.g. curiosity, unavailability or bad quality drug-of-choice)	
What are the desired effects of the substance?	
What are the unwanted (negative) effects of the substance?	
How long do the effects last?	
How many people do you estimate use this substance in your city?	
Is this new substance being combined with another substance?	
If so, with which substance?	
If combined, are any additional risks of this combination known?	

→ In case there is more than one new substance, please repeat all questions for every new substance. You can do that by copy-pasting the table above for each different substance.

   b. The emergence of a **known substance** but used for the first among (one or more of) your target group(s) in your city (e.g. GHB use among people who traditionally only used heroin)? (*Please mention only the most remarkable or worrying changes*).

( ) No, no changes were witnessed in the target group(s) of our organisation. → Please go to question 2.

( ) Yes. Please tell us:

Name of the substance	
What new group of PWUD has started to use this substance? (e.g. students, taxi drivers, asylum seekers, or any of the prompted PWUD groups)	
When did this group of PWUD start to use this substance?	
In what form is it being used by this group? (powder, pill etc.)	
How is this substance being used by this group? (Snorting, smoking, injecting, etc.)	
Why has this group started to use this substance?	
How many people in this group use this substance?	

→ In case there is more than one new target group using a known substance, please repeat all questions for every new target group you have noticed. You can do that by copy-pasting the table above for each different group.

2. In the previous year, did you witness in your target group(s) the emergence of a **new or different route of administration** of specific substances?

( ) No, no changes were witnessed in the target group(s) of our organisation. → Please go to question 3.

( ) Yes. Please tell us:

Among which of your target group(s) have you witnessed a change in their route of administration?	
Which substance(s) is/are being used differently?	
How is this substance being used now? (Snorting, smoking, injecting, etc.)	
How was this substance being used previously? (Snorting, smoking, injecting, etc.)	
Since when have you noticed this change?	
Why have PWUD changed to this route of administration?	
How many new people administer now in this way?	

→ In case there is more than one new route of administration, please repeat all questions for every new route you have noticed. You can do that by copy-pasting the table above for each new route.

3. In the previous year, did you witness in your target group(s) **new combinations of substances**?

( ) No, no changes were witnessed in the target group(s) of our organisation. → Please go to question 4.

( ) Yes. Please tell us:

Which substances are newly combined?	
Since when this change was noted?	
In which route the substances are administered?	
Which of your target group(s) combine these substances?	
Why are these substances combined? (e.g. just to get high, or for the specific effects)	
What are the desired effects of combining these substances?	
What are the unwanted (negative) effects of combining these substances?	
How many people use this new combination of substances?	

→ In case there is more than one new type of combination of substances, please repeat all questions for every new combination you have noticed. You can do that by copy-pasting the table above for each different type of combination.

Changes in the target group(s) you provide services for

4. In the previous year, did you witness any changes in the **existing target groups** you provide services for (e.g. younger, new immigrant groups)?

( ) No, no changes were witnessed in the existing target group(s) of our organisation. → Please go to question 5.

( ) Yes. Please tell us:

Which existing target group(s) changed? (e.g. users of a specific substance or any of the prompted PWUD groups)	
In what way did the target group change?	
Since when have you noticed this change?	
What are the reasons for this change?	
Have you adjusted your services in response to this change? If so, how?	

→ In case there are changes in more than one existing target group, please repeat all questions for every target group, you have noticed changes. You can do that by copy-pasting the table above for each different group.

5. In the previous year, did you start providing services for any **new group(s) of PWUD**?

( ) No, we did not. → Please go to question 6.

( ) Yes. Please tell us:

Which new group of PWUD have you started to provide services for?	
What services are provided for this new group?	
Why did you start providing services for this group?	
Is this a new group of users, or is the group new to your organisation?	
How many people do you reach with this service?	

→ In case there you started providing services for more than one new group of PWUD, please repeat all questions for every new group. You can do that by copy pasting the table above for each new group.

6. In the previous year, did you come across any new group(s) of PWUD, for whom your organisation or any other organisation are currently **not providing any services**?

( ) No, we did not. → Please go to question 7.

( ) Yes. Please tell us:

Which new group of PWUD did you come across?	
In what way this group is new?	
Since when have you noticed the emergence of this group?	
What services should be made available for this group?	

→ In case there is more than one new group, please repeat all questions for every new group you are not providing any services. You can do that by copy-pasting the table above for each group.

7. Do you have any other remark about New Drug Trends that you would like to share?

Synthetic opioids

The following questions refer to the *illicit use* of synthetic opioid in your city. These questions collect data for a European project (SO-PREP) on the preparedness of public health systems to handle a possible synthetic opioid crisis in the region. By 'synthetic opioids' we mean substances such as fentanyl, fentanyl-analogues, prescription opioids (e.g. oxycodone), and other synthetic opioids (e.g. U-47700). Please provide estimations for your city to the best of your ability.

1. Have synthetic opioids been used as illicit drugs in your city in the last 3 years?

Yes ( ) No ( ) I don't know ( )

→ If 'Yes': In which year did synthetic opioids first start being used in your city? _______

→ If 'No' or 'I don't know' → please go to **question 9**.

2. How has the use of synthetic opioids in your city changed in the last 3 years?

Decreased a lot	Decreased a little	No change	Increased a little	Increased a lot

If possible, please provide an estimate of what percentage of drug users used synthetic opioids last year, and 3 years ago:

2019: _______% 2016: _______%

3. How frequently were the following synthetic opioids used in your city last year (in 2019)?

	Frequently	Occasionally	Rarely	Never	I don't know
Fentanyl					
Fentanyl analogues, e.g. carfentanyl					
Prescription opioids, e.g. oxycodone, tramadol					
Others synthetic opioids e.g. U-47700					

4. How has the rate of overdoses involving synthetic opioids changed in the last 3 years?

Decreased a lot	Decreased a little	No change	Increased a little	Increased a lot

If possible, please provide an estimate of what percentage of overdoses involved synthetic opioids last year, and 3 years ago:

2019: _______% 2016: _______%

5. How frequently have the following synthetic opioids caused overdoses in your city last year (in 2019)?

	Frequently	Occasionally	Rarely	Never	I don't know
Fentanyl					
Fentanyl analogues, e.g. carfentanyl					
Prescription opioids, e.g. oxycodone, tramadol					
Others synthetic opioids e.g. U-47700					

6. How frequently were illicit opioids (such as heroin) adulterated with potent synthetic opioids (such as fentanyl) in your city last year (in 2019)?

Frequently	Occasionally	Rarely	Never	I don't know

7. Please describe other factors or developments that are worth mentioning regarding the prevalence and use of synthetic opioids in your city.

If your answer to question 1 was 'No':

8. How does the use of synthetic opioids in your city compare to the rest of the country?

A lot lower	A little lower	Similar	A little higher	A lot higher

Do you have any comments about this?

9. Considering the increasing trend of synthetic opioid use in Europe, what do you think are the possible explanations for having no or very little synthetic opioid use in your city in the last 3 years?

6. Civil Society Involvement

This section is a follow-up on the questions we sent out last year and aims to monitor developments in the area of civil society involvement in the field of drug policy in the last 12 months.

1. Is there any structural [more or less permanent] information and exchange mechanism between policymakers and civil society organisations in the field of drug policy in your country? (this can include exchange on drug policy development and implementation)

1a. on the national level

( ) Yes
( ) No

→**If yes,** which one of the following levels?

□ **Information**
This is a relatively low level of participation. It consists of a two-way mutual process between public authorities and CSOs of information provision and access to it.
□ **Consultation**
Through this and ad hoc mechanism through which public authorities ask CSOs for their opinion on a specific policy topic or development.
□ **Dialogue**
Dialogue entails a two-way communication mechanism built on mutual interests and potentially shared objectives to ensure a regular exchange of views.
□ **Partnership**
This mechanism articulates shared responsibilities for each step of the policymaking process: agenda-setting, policy drafting and implementation of activities. As such, this structure of participation is the most comprehensive, and it is based on co-management.

→**If no**, why not? Could you explain shortly?

1b. on the municipal level

( ) Yes
( ) No

→**If yes,** which one of the following levels?
□ **Information**
This is a relatively low level of participation. It consists of a two-way mutual process between public authorities and CSOs of information provision and access to it.
□ **Consultation**
Through this and ad hoc mechanism through which public authorities ask CSOs for their opinion on a specific policy topic or development.
□ **Dialogue**
Dialogue entails a two-way communication mechanism built on mutual interests and potentially shared objectives to ensure a regular exchange of views.

☐ **Partnership**
This mechanism articulates shared responsibilities for each step of the policymaking process: agenda-setting, policy drafting and implementation of activities. As such, this structure of participation is the most comprehensive, and it is based on co-management.
→**If no,** why not? Could you explain shortly?

2. Is <u>your organisation</u> involved in this kind of exchange?

2a. on the National Level

( ) Yes
( ) No

→ **If yes,** please describe shortly in which way. If not, please explain why not?

→ **If no,** which kind of civil society organisations are involved in these discussions? (think of treatment organisations, prevention-based organisations, abstinence-only organisations, local or national, etc.)

2b. On the municipal Level ?

( ) Yes
( ) No

→**If yes,** please describe shortly in which way. If not, please explain why not?

→**If no,** which kind of civil society organisations are involved in these discussions? (think of treatment organisations, prevention-based organisations, abstinence-only organisations, local or national, etc.)

3. Is your organisation part of any kind of CS network or national platform (in the area of harm reduction, human rights, development aid) for exchange with other CSOs on the national and/or local level?

( ) Yes
( ) No

→If yes:

3a. Please describe shortly in which kind of network your organisation is involved:

3b. Can you shortly describe the main aim and purpose of the network?

4. Please indicate how the following statements apply to the situation in your country.

The exchange between government and CS aims to:

a. inform CS on new policy developments

( ) strongly agree	( ) agree	( ) undecided	( ) disagree	( ) strongly disagree

b. collect input and knowledge from CS and grassroot level to learn more about new developments, trends and problems

( ) strongly agree	( ) agree	( ) undecided	( ) disagree	( ) strongly disagree

c. share developments, trends and problems from the field and grass root level

( ) strongly agree	( ) agree	( ) undecided	( ) disagree	( ) strongly disagree

d. discuss which kind of drug policies are effective, beneficial or harmful

( ) strongly agree	( ) agree	( ) undecided	( ) disagree	( ) strongly disagree

e. develop new strategies and approaches

( ) strongly agree	( ) agree	( ) undecided	( ) disagree	( ) strongly disagree

f. improve the access to and the quality of services (healh, social and drug-related services)

( ) strongly agree	( ) agree	( ) undecided	( ) disagree	( ) strongly disagree

5. How much do you agree with the following statements about the exchange between government and CS in your country?

a. It is organised in a **transparent** way (e.g. it is easy to follow the decision making process)

( ) strongly agree	( ) agree	( ) undecided	( ) disagree	( ) strongly disagree

b. It is organised in a **balanced** way (well represents different services, communities, worldviews)

( ) strongly agree	( ) agree	( ) undecided	( ) disagree	( ) strongly disagree

c. It is organised in a **timely** manner (e.g. CS is informed timely about any kind of new policy/development and the agenda of the meeting)

( ) strongly agree	( ) agree	( ) undecided	( ) disagree	( ) strongly disagree

d. Government officials are easily **approachable** for CSOs (e.g. they respond to emails/phone calls)

( ) strongly agree	( ) agree	( ) undecided	( ) disagree	( ) strongly disagree

e. Decision makers are represented in the appropriate **level** (e.g. those who make deicisions are involved)

( ) strongly agree	( ) agree	( ) undecided	( ) disagree	( ) strongly disagree

f. The government is **open** to civil society initiatives (e.g. civil society initiatives are easily taken up by government)

( ) strongly agree	( ) agree	( ) undecided	( ) disagree	( ) strongly disagree

g. Adequate **funding** is provided (e.g. there is public funding for advocacy work)

( ) strongly agree	( ) agree	( ) undecided	( ) disagree	( ) strongly disagree

h. Civil society input is **heard** and taken into account when it comes to decision making

( ) strongly agree	( ) agree	( ) undecided	( ) disagree	( ) strongly disagree

i. Civil society can speak openly and frankly and criticise **without facing repercussions** or budget cuts

( ) strongly agree	( ) agree	( ) undecided	( ) disagree	( ) strongly disagree

5. Is your organisation cooperating with and contributing to the national EMCDDA Reporting or other types of data reporting in your country?

( ) Yes
( ) No

→ **If yes,** could you briefly describe this cooperation?

→ **If no,** could you please describe why not?

6. Is there anything else concerning civil society involvement what you would like to share with us?

7. COVID-19

This is a special session added to the 2020 Monitoring to address if/how the Covid-19 pandemic has influenced harm reduction services.

1. Has the Covid-19 pandemic affected daily practices in your harm reduction program/ service?

   ( )Yes ( ) No

2. What challenges did your harm reduction service face during the pandemic? Please check all that apply.

   ( ) We closed the facility
   ( ) We reduced opening hours/days
   ( ) PWUD were not allowed access due to lockdown
   ( ) Limitation in harm reduction supplies (syringes, sterilisation equipment)
   ( ) Limited access to protective equipment for staff and clients (surgical masks, sanitising materials)
   ( ) Reduction in types of harm reduction services available
   ( ) We adapted our service (masks, distance, Plexiglas, limited controlled access, etc.) to ensure the continuation of activities
   ( ) Others/comments

3. What positive changes or innovations in harm reduction services occurred at your organisation? Please check all that apply.

   ( ) Improvement in OST services
   ( ) Increased length of prescriptions and take-homes
   ( ) New phone or telemedicine services for OST
   ( ) increased interest and enrolment in OST
   ( ) home delivery for OST
   ( ) New forms of OST available
   ( ) Improvement regarding access to housing and shelters
   ( ) Increased or started Naloxone distribution
   ( ) Added new outreach services
   ( ) Education services on COVID and hygiene/safety for PWUD

Other:

4. Do you think that the pandemic was an opportunity to implement innovative harm reduction service practices?

5. Which are the main difficulties that PWUD have to face in your country during the coronavirus pandemic? Please rate how important these difficulties are.

	Very problematic	Problematic	Somewhat problematic	Not a problem
Limited access to drugs				
Adulterated/low-quality drugs				
Increased drug prices				
Limited access to OST				
Limited access to DCRs				
Limited access to drug checking				
Limited access to medical services				
Limited access to housing				
Difficulties with the police when being in the street				
Social isolation				
Increase in mental health disorders				

Others, please describe below:

6. The COVID pandemic has been reported to increase the risk of overdose for vulnerable PWUD, due to more people using alone, less access to naloxone, increased respiratory risk, increased adulterated substances, etc. Have you noted an increase in OD in your region during the pandemic?
   ( ) Yes
   ( ) No
   ( ) I don't know

7. Was there a government response to protect PWUD and harm reduction professionals during the pandemic
   ( ) No specific response
   ( ) Yes, please describe below

8. How do you score the response of your government regarding pandemic prevention for people who use drugs?

( ) Excellent	( ) Above average	( ) Average	( ) Below average	( ) Very poor

9. What role did CSO's play in advocating or increasing services for PWUD during the pandemic?

10. Other comments on harm reduction and impact on PWUD of the COVID pandemic?
